# Physical exercise and risk of common mental disorders among higher education students: A national prospective study

**DOI:** 10.1371/journal.pone.0353787

**Published:** 2026-08-19

**Authors:** Michael Grasdalsmoen, Erlingur Johannsson, Mari Hysing, Børge Sivertsen

**Affiliations:** 1 Department of Sport, Food and Natural Sciences, Western Norway University of Applied Sciences, Bergen, Norway; 2 Center of Sport and Health Sciences, School of Education, University of Iceland, Reykjavik, Iceland; 3 Department of Psychosocial Science, Faculty of Psychology, University of Bergen, Bergen, Norway; 4 Department of Health Promotion, Norwegian Institute of Public Health, Bergen, Norway; 5 Department of Research & Innovation, Helse Fonna HF, Haugesund, Norway; Chulalongkorn University Faculty of Psychology, THAILAND

## Abstract

**Background:**

Physical exercise is associated with better mental health and lower psychological distress, yet few studies have examined its relationship with diagnostically defined common mental disorders (CMD) in young adults. This study investigated prospective associations between multiple dimensions of physical exercise and subsequent CMD among students in higher education.

**Methods:**

Data were drawn from the Students’ Health and Wellbeing Study (SHOT2022), a national survey of Norwegian higher education students (n = 53,362; age 18−35 years). Physical exercise frequency, intensity, duration, and total weekly hours were assessed at baseline. CMD, defined as major depressive episode (MDE) or generalized anxiety disorder (GAD) during the past 30 days or 12 months, was assessed one year later using the self-administered Composite International Diagnostic Interview (CIDI 5.0) (n = 10,460). Associations between exercise indicators and CMD were examined using inverse probability weighted Poisson regression models, yielding risk ratios (RR) and 95% confidence intervals (CI), stratified by sex and examined across two age groups (18−24 and 25−35 years), and sequentially adjusted for sociodemographic variables and baseline psychological distress (HSCL-25).

**Results:**

Lower levels of physical exercise were consistently associated with higher relative risk of subsequent CMD. Compared with daily exercisers, those who seldom or never exercised had higher adjusted RR of CMD (women: RR = 1.15, 95% CI 1.04–1.28; men: RR = 1.33, 95% CI 1.06–1.65). Below-recommendation activity levels were likewise linked to elevated risk. Weekly exercise hours were inversely related to CMD up to ten hours per week, with evidence of a curvilinear association among women. Age-stratified analyses suggested somewhat stronger and more clearly graded associations among students aged 18–24 years; however, formal interaction analyses did not support meaningful effect modification by age group.

**Conclusions:**

These findings support a prospective association between regular physical exercise and a lower likelihood of depression and anxiety among students in higher education. The findings further suggest that consistent engagement in exercise, rather than high intensity or extreme volumes, may be particularly important, underscoring the value of promoting attainable and sustainable activity patterns in this population.

## Introduction

Common mental disorders (CMD), encompassing major depressive and anxiety disorders, are among the leading causes of disability worldwide in young adults [1], and the transition into higher education is a particularly vulnerable period for onset and persistence of clinically significant disorders. Large international studies from the World Mental Health-International College Student (WMH-ICS) initiative indicate substantial 12-month prevalence and impairment among college students [2–4]. Students in tertiary institutions frequently report elevated levels of symptoms compared to non-student peers [5–7], underscoring the critical importance of identifying modifiable protective factors during this developmental stage. At the same time, physical inactivity remains pervasive among young adults [8], despite public health recommendations promoting regular moderate‐to‐vigorous physical activity (MVPA). The World Health Organization 2020 guideline recommends at least 150–300 minutes of moderate-intensity aerobic activity per week and muscle strengthening activity at least two days a week for adults [9]. Yet, many higher-education students fall short of these thresholds [10].

Accumulating research supports the hypothesis that regular physical exercise is associated with better mental health, including lower risk of CMD. Recent large-scale systematic and umbrella reviews show that higher levels of physical activity are associated with lower risk of depression and anxiety in both observational studies [11] and intervention studies [12]. Although less is known about the minimal level of physical activity needed for mental health benefits, a meta-analysis of 15 prospective adult studies found that even activity levels below current recommendations were associated with lower risk of depression [13].

Associations between physical activity and mental health have also been shown in student populations [14,15]. In a large national survey of Norwegian higher education students, we found that higher frequency, intensity, and duration of exercise were each inversely related to several indicators of mental health problems in a clear dose-response pattern, with frequency emerging as the strongest correlate [16].

Similar patterns have been observed in children and adolescents. A nationwide cohort study found that higher levels of physical fitness in childhood and adolescence were associated with lower subsequent risk of mental disorders [17]. Similarly, an updated systematic review and meta-analysis reported beneficial effects of physical activity interventions across multiple mental health domains, including reductions in symptoms of anxiety and depression and improvements in overall psychological well-being among children and adolescents [18].

Despite this broad evidence base, several important gaps remain. First, many prior investigations have relied on screening instruments rather than diagnostic assessments of CMD, which reduces clinical relevance. Second, relatively few studies have examined prospective associations between physical exercise and common mental disorders using standardized diagnostic instruments. This distinction is important, as such outcomes may better capture clinically significant conditions and thus have greater relevance for prevention and public health. Third, few studies have accounted adequately for baseline psychological distress or applied analytic approaches that address selection processes, such as inverse probability weighting, which can mitigate bias arising from differential follow‐up. Furthermore, given the substantial overlap and shared etiological features of depressive and anxiety disorders [19,20], often conceptualized jointly as internalizing disorders, analysing them jointly as CMD provides a broader and clinically relevant endpoint than considering each disorder separately.

The present prospective study addresses these gaps by drawing on baseline data from SHoT2022 and a one-year self-administered follow-up diagnostic assessment of current CMD using the Composite International Diagnostic Interview (CIDI 5.0). We examine prospective associations between exercise frequency, intensity, duration, and total weekly hours, as well as adherence to WHO MVPA recommendations, and subsequent CMD. To strengthen the robustness of the prospective analyses, we apply inverse probability weighting to reduce bias from differential follow-up and adjust for sociodemographic characteristics and baseline psychological distress. By combining a prospective design, diagnostically defined outcomes, and multiple dimensions of physical exercise, the study provides a clinically relevant and methodologically robust assessment of the association between exercise and CMD in higher education students.

## Methods

### Study design, participants and setting

This study is based on data from the Students’ Health and Wellbeing Study (SHOT), a national survey targeting students in higher education in Norway. The SHOT survey has been conducted every four years since 2010, with the most recent wave, SHOT2022, administered between February 8 and April 19, 2022. SHOT2022 collected comprehensive information on mental and somatic health, lifestyle, social relationships, and financial stressors. The detailed methodology of the SHOT study has been described previously [21].

SHOT2022 was distributed digitally via a web-based questionnaire to all full-time students in Norwegian higher education institutions, including those studying abroad. Invitations were sent via email and SMS, supported by coordinated efforts from student welfare organizations and educational institutions. Of the 169,572 students invited, 59,544 participated (response rate 35.1%). For the present study, we included a subsample of 53,362 students who were aged 18–35 years, enrolled full-time, and had complete data on physical exercise indicators.

**Fig 1:**
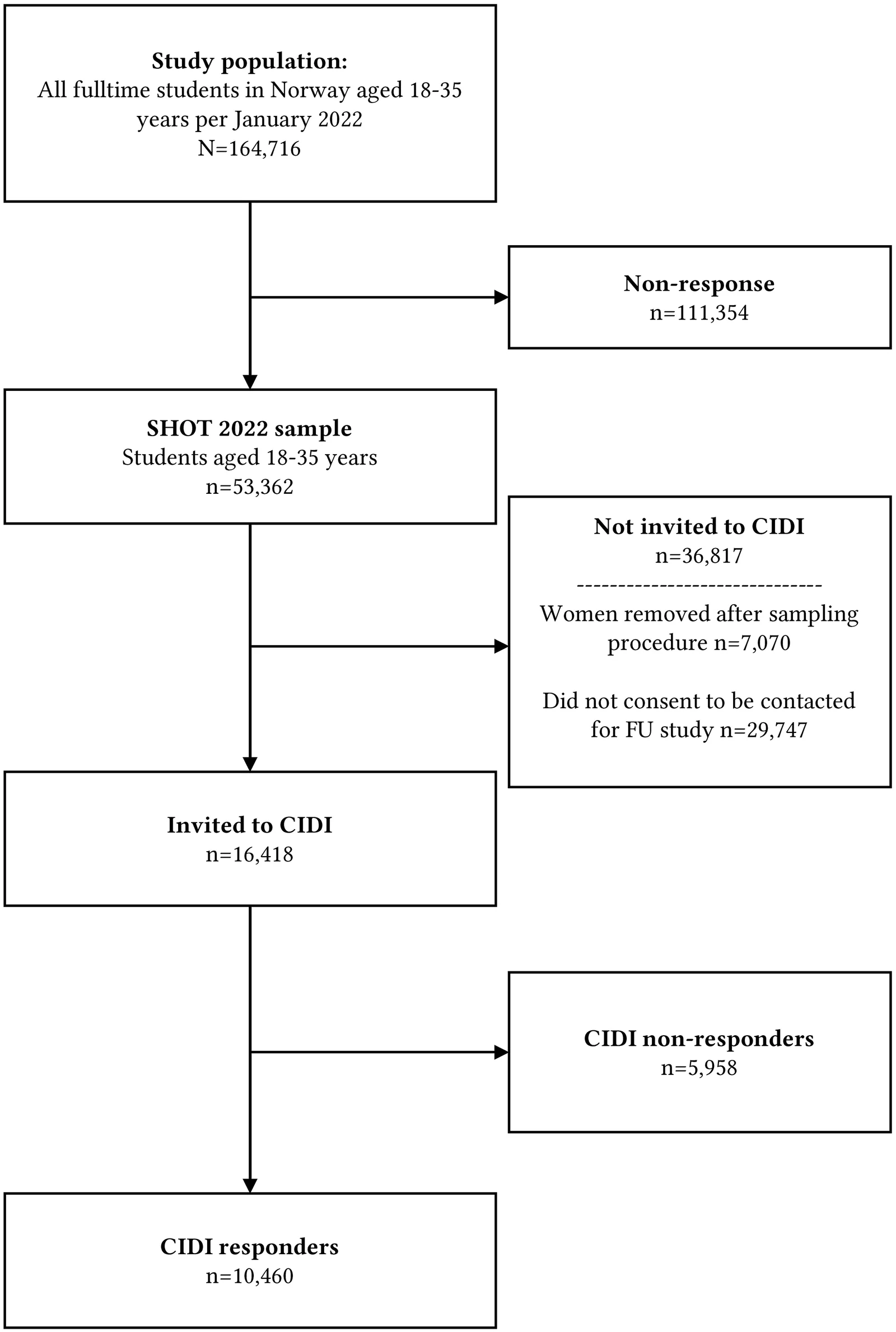
Flowchart of study participants.

To assess subsequent CMD, a follow-up diagnostic study (the CIDI study) was conducted approximately one year after the main survey, from January 24 to February 6, 2023. The follow-up interval was determined by the design of the SHOT diagnostic follow-up study and was not specifically tailored to the present research question. Of the 26,311 students who indicated willingness to be recontacted, 16,418 were invited to participate, with oversampling of men to counteract sex imbalance. Ultimately, 10,460 students completed at least one diagnostic module, corresponding to 63.7% of those invited and 17.6% of the baseline SHOT2022 sample. A flowchart of participation is shown in Fig 1.

To account for known differences in physical activity and mental health across the student age span, analyses were additionally stratified into two age groups (18–24 and 25–35 years). This categorization reflects the age distribution of the SHOT cohort and common distinctions between younger and older students in higher education

### Instruments

#### Sociodemographic information.

Age and sex were obtained from the Norwegian national identity number registry. Other background variables were self-reported in SHoT2022, including civil status (single, cohabiting, married), migration background (self or parental birth outside Norway), parental education (categorized in three levels), and whether the respondent had children of their own (student parental status), and accommodation status (dichotomized as living alone vs. living with others).

### Physical exercise

The students were first presented with the following brief definition of physical exercise: *“With exercise we mean that you, for example, go for a walk, go skiing, swim, or take part in a sport.”* Physical exercise was then assessed through three questions capturing average frequency, intensity, and duration of activity [22]:

1. “How frequently do you exercise?” (Never, Less than once a week, Once a week, 2–3 times per week, Almost every day);
2. “If you exercise as frequently as once or more times a week: How hard do you push yourself?” (I take it easy without breaking into a sweat or losing my breath; I push myself so hard that I lose my breath and break into a sweat; I push myself to near exhaustion); and
3. “How long does each session last?” (Less than 15 minutes, 15-29 minutes, 30 minutes to 1 hour, More than 1 hour).

This three-item questionnaire has previously been used in the large population-based Nord-Trøndelag Health Study (HUNT) [22,23]. In the present study, the response options “Never” and “Less than once a week” were combined for the frequency item to form the reference category. Similarly, “Less than 15 minutes” and “15-29 minutes” were combined for the duration item. Detailed information on the physical exercise measures in the SHOT studies has been published elsewhere [10]. Previous validation studies [22,23] have demonstrated moderate correlations between these questionnaire responses and direct measurement of VO_2_max during maximal work on a treadmill (r = 0.43[frequency], r = 0.40 [intensity] and r = 0.31 [duration]), with ActiReg [24,25], an instrument that measures PA and energy expenditure (EE), and with the International Physical Activity Questionnaire [26]. Based on the World Health Organization (WHO) recommendations [27] that adults (≥18 years) should engage in at least 30 minutes of moderate-to-vigorous physical activity (MVPA) on five or more days per week (equivalent to ≥150 minutes per week), a dichotomous recommendation variable was created using participants’ responses to the three exercise items (MVPA ≥150 min/week: students reporting “Almost every day” on the frequency item, “I push myself so hard that I lose my breath and break into a sweat” on the intensity item, and “30 minutes to 1 hour” or “More than 1 hour” on the duration item).

Finally, respondents who did not answer “never” to the exercise frequency item also reported how many hours per week they trained (response options: 0–40 hours). For the present analyses, this variable was treated as a continuous measure of total weekly hours of exercise, with values above 10 collapsed into 10 hours per week to avoid sparse data in the upper range.

In line with the SHoT questionnaire wording, we use the term *physical exercise* to refer to the specific frequency, intensity and duration measures used in this study. This construct is narrower than the broader WHO concept of physical activity.

### Psychological distress: HSCL-25

Baseline symptoms of anxiety and depression (SHOT2022) were assessed using the 25-item Hopkins Symptom Checklist (HSCL-25), a widely used instrument for measuring psychological distress in population-based studies [28]. Each item is rated on a four-point Likert scale ranging from 1 (not at all) to 4 (extremely), and the mean of all items is used to derive an overall distress score. For the present analyses, psychological distress was dichotomized using sex-specific cut-offs recently validated in a Norwegian student population: ≥ 1.96 for men and ≥2.20 for women. These thresholds have been shown to provide a better balance between sensitivity and specificity than the traditional cut-off of 1.75 [29]. The binary HSCL-25 variable was included in the fully adjusted models (Model 3) to control for baseline distress that could confound the association between physical exercise and subsequent CMD.

### Common Mental Disorder (CMD): CIDI 5.0

Current common mental disorder (CMD), comprising major depressive episode (MDE) and/or generalized anxiety disorder (GAD), was assessed using the Norwegian version of the Composite International Diagnostic Interview (CIDI) 5.0, a self-administered, web-based diagnostic instrument developed for the WHO World Mental Health Surveys [30]. The CIDI assesses 30-day, 12-month, and lifetime prevalence of mental disorders according to DSM-5 criteria [31]. CIDI has demonstrated acceptable concordance with clinican-administered diagnostic interviews, including the Schedules for Clinical Assement in Neuropsychiatry (SCAN) [32]. The Norwegian version was developed following the official WHO translation and adaptation procedures [33].

For the present study, CMD was defined as meeting DSM-5 diagnostic criteria for either MDE or GAD within the past 30 days or 12 months at the CIDI follow-up assessment. Participants who reported only lifetime (but not 12-month or 30-day) episodes were excluded, ensuring that all included cases reflected active or recent disorders relative to baseline SHoT2022 data. A combined binary variable (CMD = 1, no CMD = 0) indicated the presence of either current MDE or GAD, while participants with past-only diagnoses were coded as missing. As diagnostic CMD was not assessed at baseline, participants with prevalent CMD at baseline could not be excluded. The outcome at follow-up therefore reflects a combination of incident, persistent, and recurrent cases.

### Statistical analyses

All descriptive analyses (Table 1) were conducted on unweighted data, consistent with standard conventions for reporting sample characteristics [34,35]. To address potential bias due to differential response, all regression analyses applied inverse probability weights (IPW) [36]. Such bias may occur if participation in the CIDI follow-up is systematically related to sociodemographic or clinical characteristics. Response probabilities were estimated using a logistic regression model including baseline sociodemographic variables and HSCL-25 scores but excluding the physical exercise indicators (exposures) to avoid overadjustment. Each participant was weighted by the inverse of their predicted probability of response, so that the weighted sample more closely reflects the full SHOT2022 population.

**Table 1:** Sociodemographic and clinical characteristics in 2022 of the CIDI responders, CIDI non-responders and the overall SHOT2022 sample.

Characteristic	CIDI responders(n = 10,460)	CIDI non-responders(n = 5,958)	p-value^#^	SHOT2022* (n = 53,362)	p-value^#^
Age, mean (SD)	24.03 (3.28)	23.97 (3.24)	.24	23.98 (1.85)	.14
Sex, % (n)			.97		<.001
Women	70.6 (7,386)	70.4 (4,196)		66.4 (35,423)	
Men	29.4 (3,074)	29.6 (1,762)		33.6 (17,939)	
Marital status, % (n)			.16		.70
Single	51.2 (5,359)	50.3 (2,994)		51.0 (27,197)	
Boy-/girlfriend	22.4 (2,343)	23.7 (1,414)		22.8 (12,152)	
Cohabitant	22.7 (2,375)	22.5 (1,340)		22.6 (12,058)	
Married/registered partner	3.3 (345)	3.0 (178)		3.1 (1,667)	
Self and/or parent(s) born abroad, % (n)			.17		.34
Born in Norway	81.2 (8,491)	80.4 (4,792)		80.1 (43,052)	
Born outside Norway	10.0 (1043)	10.9 (651)		10.4 (5,541)	
Maternal education, % (n)			.04		.50
Primary	4.4 (457)	5.3 (313)		4.5 (2,407)	
Secondary	27.3 (2,857)	27.6 (1,646)		27.6 (14,707)	
College/university	65.6 (6,858)	64.2 (3,827)		64.3 (34,326)	
Paternal education, % (n)			.02		.52
Primary	5.7 (599)	6.8 (406)		6.0 (3,182)	
Secondary	35.2 (3,678)	34.9% (2,078)		35.1 (18,735)	
College/university	54.2 (5,674)	52.8 (3,145)		53.3 (28,446)	
HSCL-25, Mean (SD)	1.88 (0.61)	1.89 (.61)	.30	1.86 (0.59)	.01
*Missing,% (n)*	*0.2 (17)*	*0.3 (24)*		*0.4 (214)*	


SHOT2022: Students’ Health and Wellbeing Study 2022; CIDI: Composite International Diagnostic Interview; HSCL-25 Hopkins Symptoms Checklist – 25 items version* grand mean for the SHOT2022 sample aged 18–35; ^**#**^ compared with the CIDI responders group (p-values based on Chi-squared test (categorical variables) or t-test (continuous variables).

Associations between physical exercise indicators and subsequent CMD were examined using Poisson regression with robust standard errors, yielding risk ratios (RR) and 95% confidence intervals (CI). A log link function was specified, allowing direct estimation of relative risks rather than odds ratios, in line with Zou’s modified Poisson approach for binary outcomes [37]. Because both weighting (IPW) and covariate adjustment were applied, the analyses can be considered “doubly robust”, meaning that estimates remain valid if either the weighting model or the outcome model is correctly specified [38]. Potential effect modification by sex and age group was formally assessed by including interaction terms between each physical exercise indicator and sex, and between each exercise indicator and age group, within the modified Poisson regression framework. Evidence of interaction was evaluated using Wald tests of the interaction terms. As interaction analyses indicated differences by sex, all main models were subsequently stratified by sex. Age-stratified analyses were conducted for descriptive purposes; however, the overall age group × exercise frequency interaction was not statistically significant. To address confounding and clarify temporal direction, a three-step adjustment strategy was applied: Model 1: unadjusted; Model 2: adjusted for sociodemographic covariates (age, immigrant background, parental education, student parental responsibility, and relationship and accommodation status); and Model 3: additionally adjusted for baseline psychological distress (HSCL-25). Baseline psychological distress (HSCL-25) was included in the fully adjusted model to account for potential confounding by pre-existing mental health. However, HSCL-25 may also lie on the causal pathway between physical exercise and subsequent CMD. Accordingly, models with and without adjustment for HSCL-25 are presented to distinguish between total associations (Model 2) and more conservative estimates that account for baseline distress (Model 3). While the exercise indicators are conceptually related and moderately correlated, they were not combined or mutually adjusted in the main analyses, as the primary aim was to examine their independent associations with CMD.

To further examine weekly exercise volume, supplementary analyses were conducted modelling weekly exercise hours both categorically and as a continuous exposure. Weekly exercise hours were capped at 10 or more hours per week to limit the influence of extreme values. In the continuous models, weekly exercise hours were entered as a linear term, and a quadratic term (hours²) was included to assess potential non-linearity. Evidence of non-linearity was evaluated based on the statistical significance and direction of the quadratic term. Predicted relative risks were derived from these models using 0 hours per week as the reference and presented graphically with 95% confidence intervals.

Sensitivity analyses were conducted using alternative outcome definitions, including separate models for 30-day and 12-month generalized anxiety disorder (GAD) and major depressive episode (MDE). These analyses were specified using the same modelling framework and adjustment strategy as the primary analyses and were used to assess the robustness of the findings across diagnostic categories and time frames. As a supplementary analysis, mutually adjusted models including exercise frequency, intensity, and duration simultaneously were estimated; these were based on a reduced subsample with complete data on all exercise dimensions. All analyses were performed in IBM SPSS Statistics 30 [39] and R version 4.5.3 in RStudio 2026.01.2 + 418.

### Ethics

The study adhered to the STROBE reporting guidelines. Ethical approval for the SHoT2022 study was obtained from the Regional Committee for Medical and Health Research Ethics in Western Norway (reference no. 326437). All participants provided electronic informed consent after receiving detailed information about the study’s aims and procedures.

## Results

### Sample characteristics and representativeness

Table 1 summarizes key sociodemographic and clinical characteristics of participants in the CIDI follow-up study compared with the full SHOT2022 sample. The CIDI responder sample in.cluded a higher proportion of womwn than the overall SHOT2022 sample. Otherwise, the demographic and clinical characteristics were broadly similar across the samples. Baseline psychological distress (HSCL-25) scores were marginally lower among responders than non-responders (Cohen’s d = 0.03), suggesting minimal response bias.

### Distribution of physical exercise indicators

Table 2 shows the sex-stratified distribution of exercise frequency, intensity, duration, and weekly exercise hours. For both women and men, the most common exercise frequency was 2–3 times per week (38.0% and 33.8%, respectively), followed by 4–5 times per week. Daily exercise was reported by 17.5% of women and 20.5% of men, while around 8–10% reported never or seldom exercising. Most participants reported moderate exercise intensity (71.8% of women and 69.1% of men). High-intensity exercise was more commonly reported among men (12.8%) than women (5.4%), whereas a greater proportion of women reported low-intensity exercise. Regarding duration, the majority of women reported exercising 30–60 minutes per session (54.7%), while men more frequently reported sessions lasting more than 60 minutes (47.5%). Short durations (<30 minutes) were relatively uncommon in both sexes.

**Table 2:** Distribution of physical exercise indicators, by sex.

	Females			Males		
Variable	Category	n	%	Category	n	%
Exercise frequency	Never/seldom	366	7.8	Never/seldom	190	10.1
	1 time/week	544	11.6	1 time/week	228	12.1
	2-3 times/week	1781	38.0	2-3 times/week	638	33.8
	4-5 times/week	1176	25.1	4-5 times/week	444	23.5
	Every day	820	17.5	Every day	386	20.5
Exercise intensity	Easy	1072	22.9	Easy	341	18.1
	Moderate	3364	71.8	Moderate	1304	69.1
	High	251	5.4	High	241	12.8
Exercise duration	<15 min	58	1.2	<15 min	31	1.6
	15-29 min	524	11.2	15-29 min	176	9.3
	30-60 min	2564	54.7	30-60 min	784	41.6
	>60 min	1541	32.9	>60 min	895	47.5
Weekly exercise hours	0 h	453	6.9	0 h	195	7.2
	1-2 h	1653	25.2	1-2 h	658	24.5
	3-4 h	2154	32.8	3-4 h	672	25.0
	5-6 h	1206	18.4	5-6 h	500	18.6
	7-9 h	685	10.4	7-9 h	360	13.4
	10 + h	416	6.3	10 + h	307	11.4

Note: Percentages based on available cases for each variable. Exercise intensity and duration were assessed among participants reporting regular exercise.

The distribution of weekly exercise hours was broadly similar across sexes, with the largest proportion reporting 3–4 hours per week (32.8% of women and 25.0% of men). Men were more likely to report higher exercise volumes, particularly ≥10 hours per week (11.4% vs. 6.3%), whereas the proportion reporting no exercise was comparable between sexes (approximately 7%).

### Associations between physical exercise and CMD

In the weighted CIDI sample, most indicators of physical exercise showed inverse associations with the RR of CMD one year later. As shown in Table 3, students who exercised infrequently, with low intensity or short duration, or who did not meet the WHO recommendation of ≥150 minutes per week generally had higher RR of CMD compared with their more active peers. Adjustment for sociodemographic factors (Model 2) had only modest impact on the estimates, while further adjustment for baseline psychological distress (Model 3) led to partial attenuation. Formal interaction analyses showed a statistically significant sex × exercise frequency interaction (Wald χ² = 14.91, df = 4, p = .005). Category-specific interaction terms indicated that sex differences were most evident at the two lowest exercise frequency levels: never/seldom exercise (p = .044) and exercise once per week (p = .034).

**Table 3:** Estimated prevalence and relative risk (RR) of common mental disorder (CMD) among female and male students, by physical exercise indicator.

FEMALES	CMD % (IPW-weighted and age-adjusted))	RR (Model 1)	RR (Model 2)	RR (Model 3)
**Exercise frequency**				
Never/seldom	59.9%	1.44 (1.31-1.58)	1.38 (1.23-1.56)	1.15 (1.04-1.28)
1 time/week	51.8%	1.25 (1.12-1.38)	1.21 (1.07-1.37)	1.08 (0.98-1.21)
2-3 times/week	46.0%	1.10 (1.01-1.20)	1.11 (1.01-1.23)	1.06 (0.98-1.16)
4-5 times/week	45.9%	1.10 (1.01-1.21)	1.12 (1.01-1.25)	1.09 (0.99-1.20)
Every day (ref)	41.6%	1.00	1.00	1.00
**Exercise intensity (average)**				
Easy (no sweat/out of breath)	56.1%	1.44 (1.25-1.67)	1.40 (1.18-1.66)	1.31 (1.13-1.52)
Hard (sweat/out of breath)	44.1%	1.14 (0.99-1.32)	1.13 (0.96-1.34)	1.17 (1.01-1.35)
Almost to exhaustion (ref)	38.7%	1.00	1.00	1.00
**Exercise duration (average)**				
<15 minutes	61.6%	1.34 (1.13-1.59)	1.30 (1.03-1.63)	1.18 (0.99-1.42)
15-29 minutes	55.0%	1.19 (1.10-1.30)	1.17 (1.06-1.29)	1.06 (0.97-1.15)
30-60 minutes	45.5%	0.99 (0.93-1.05)	0.98 (0.91-1.05)	0.98 (0.92-1.05)
>1 hour (ref)	45.8%	1.00	1.00	1.00
**Meets MVPA ≥150 min/week**				
Below recommendation	50.8%	1.25 (1.18-1.33)	1.21 (1.13-1.30)	1.10 (1.03-1.17)
Above recommendation (ref)	40.4%	1.00	1.00	1.00
**MALES**				
**Exercise frequency**				
Never/seldom	45.2%	1.94 (1.59-2.38)	1.85 (1.45-2.37)	1.33 (1.06-1.65)
1 time/week	36.9%	1.61 (1.29-2.01)	1.77 (1.37-2.29)	1.31 (1.03-1.66)
2-3 times/week	29.6%	1.29 (1.06-1.57)	1.24 (0.98-1.56)	0.98 (0.79-1.21)
4-5 times/week	27.6%	1.22 (0.98-1.51)	1.24 (0.97-1.59)	1.02 (0.81-1.27)
Every day (ref)	23.1%	1.00	1.00	1.00
**Exercise intensity (average)**				
Easy (no sweat/out of breath)	38.2%	1.34 (1.08-1.67)	1.23 (0.96-1.57)	1.16 (0.94-1.44)
Hard (sweat/out of breath)	28.6%	1.02 (0.83-1.24)	0.96 (0.77-1.20)	1.03 (0.85-1.24)
Almost to exhaustion (ref)	27.9%	1.00	1.00	1.00
**Exercise duration (average)**				
<15 minutes	42.2%	1.59 (1.10-2.30)	1.62 (1.05-2.51)	1.67 (1.17-2.39)
15-29 minutes	40.4%	1.53 (1.28-1.84)	1.49 (1.20-1.86)	1.22 (1.01-1.48)
30-60 minutes	32.1%	1.23 (1.07-1.40)	1.21 (1.03-1.41)	1.10 (0.96-1.26)
>1 hour (ref)	25.7%	1.00	1.00	1.00
**Meets MVPA ≥150 min/week**				
Below recommendation	35.1%	1.48 (1.29-1.69)	1.41 (1.20-1.65)	1.17 (1.01-1.34)
Above recommendation (ref)	23.5%	1.00	1.00	1.00

Note: Model 1: unadjusted. Model 2: adjusted for sociodemographic covariates. Model 3: additionally adjusted for baseline psychological distress (HSCL-25). MVPA = moderate-to- vigorous physical activity.

A clear dose-response pattern was observed for exercise frequency in both sexes. Among women, compared with those exercising daily, students who seldom or never exercised had a 44% higher unadjusted CMD risk (Model 1 RR = 1.44; 95% CI: 1.31–1.58), which decreased but remained significant after full adjustment (RR = 1.15; 1.04–1.28). Those exercising once or 2−3 times per week also showed modestly elevated risks (Model 3 RR = 1.08; 0.98–1.21 and 1.06; 0.98–1.16, respectively). Among men, the corresponding gradient was even steeper: compared with daily exercisers, those reporting seldom or never activity had nearly double the unadjusted risk (RR = 1.94; 1.59–2.38), which remained significant after full adjustment (RR = 1.33; 1.06–1.65).

For exercise intensity, women who exercised at an easy level had a 31% higher fully adjusted RR of CMD (RR = 1.31; 1.13–1.52) compared with those exercising “almost to exhaustion.” Among men, no significant adjusted association emerged for intensity. For exercise duration, no consistent pattern was observed among women after adjustment. Among men, shorter sessions were associated with higher CMD risk. Compared with sessions lasting more than one hour, the fully adjusted RRs were 1.67 (1.17–2.39) for less than 15 minutes and 1.22 (1.01–1.48) for 15−29 minutes. Finally, meeting the WHO recommendation was associated with lower CMD risk in both sexes. Among women, those below the guideline had a 25 percent higher unadjusted risk (RR = 1.25; 1.18–1.33), which remained significant after adjustment (RR = 1.10; 1.03–1.17). In men, the corresponding fully adjusted RR was 1.17 (1.01–1.34).

### Association between weekly exercise and CMD

**Fig 2:**
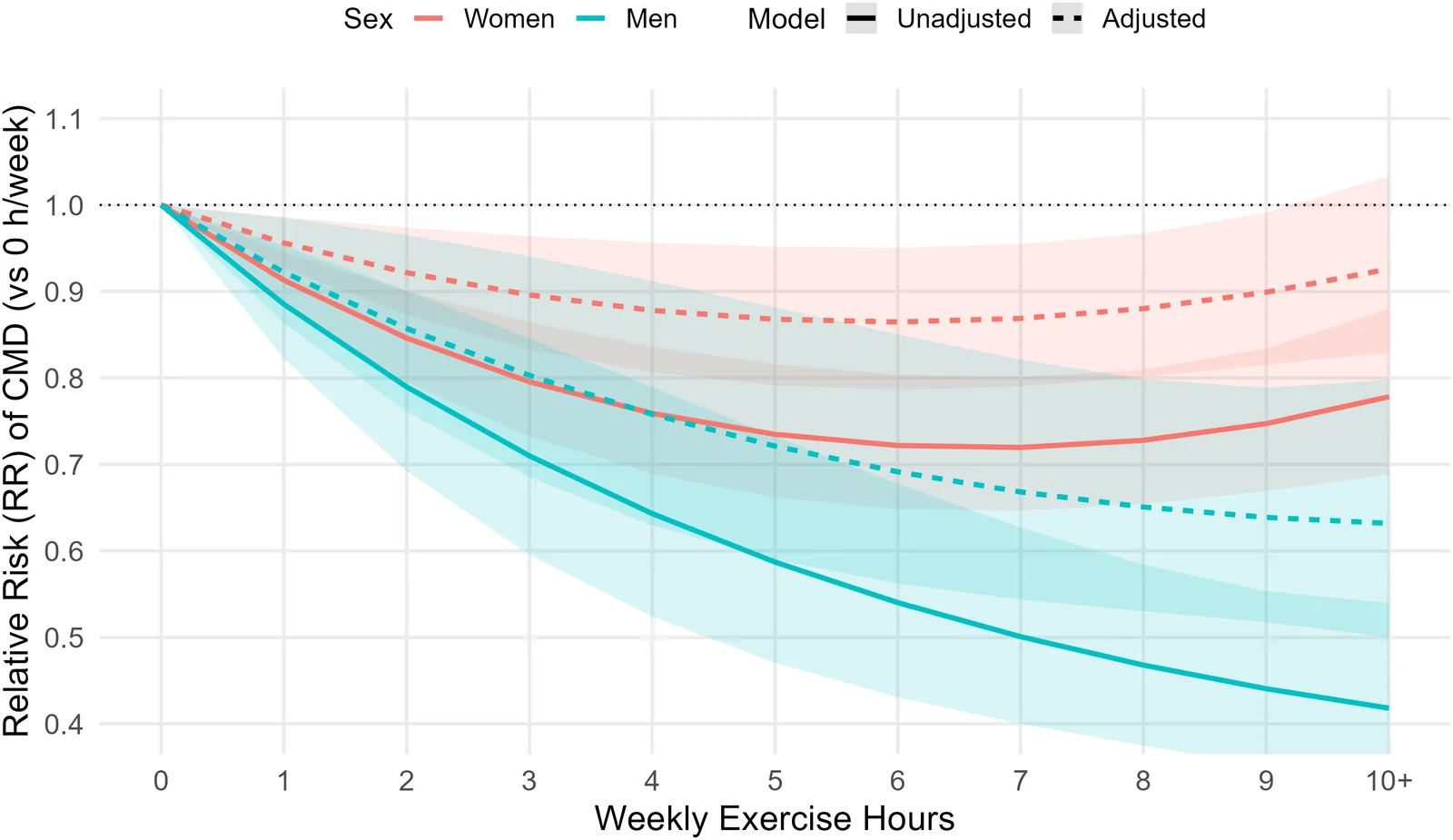
Relative risk of CMD by weekly exercise hours in women and men. Lines show predicted relative risks from modified Poisson regression models with robust standard errors, using 0 hours per week as the reference. Weekly exercise hours were capped at 10 hours per week and modelled using linear and quadratic terms. Solid lines indicate unadjusted models and dashed lines indicate adjusted models. Shaded areas represent 95% confidence intervals.

Fig 2 illustrates the relation between weekly exercise hours and CMD relative risk one year later. Both unadjusted and adjusted curves showed a graded decline in CMD risk up to about ten hours per week, followed by a plateau. Among men, the unadjusted trend showed a sharp and nearly linear reduction in RR of CMD across increasing weekly hours, with RR approximately 40–50 percent lower among those exercising ten or more hours per week compared with inactive peers. Adjustment for sociodemographic factors and baseline distress attenuated this pattern, although the overall shape remained similar. Among women, the association was more curvilinear. RR of CMD declined steeply between zero and about five hours per week and then levelled off gradually. At ten or more hours per week, RR was about 25–30 percent lower than among inactive women. Adjustment led to noticeable attenuation, particularly at higher levels of exercise, where the adjusted curve showed a less pronounced decline and a clearer plateau compared with the unadjusted estimates.

Overall, while the magnitude of the associations differed between models, the inverse pattern and the evidence of diminishing returns at higher activity levels were consistent across models. At approximately 5-6 hours of weekly exercise among women, the estimated RR reached its lowest lwvel before gradually increasing, whereas among men the RR continued to decline across higher weekly exercise volumes. The associations were substantially attenuated in the adjusted models (Fig 2). A formal test of nonlinearity supported this pattern: the quadratic term was statistically significant among women (p < .05), indicating a curvilinear association, whereas no statistically significant evidence of non-linearity was observed among men.

Supplementary analyses using categorical weekly exercise hours showed the highest CMD prevalence among inactive students, with a graded decline across increasing activity levels, particularly among men (Supplementary Tables S6 and S8 in S1 File). In continuous models, weekly exercise hours were inversely associated with CMD in both sexes. Formal tests of non-linearity supported a curvilinear association among women, with diminishing benefit at higher activity levels, whereas no statistically significant evidence of non-linearity was observed among men (Supplementary Table S8 in S1 File). In mutually adjusted models including exercise frequency, intensity, and duration simultaneously, exercise frequency remained the most consistent predictor of CMD, whereas independent associations for duration were weak and inconsistent. Among women, low exercise intensity also remained associated with higher CMD risk (Supplementary Table S7 in S1 File).

### Age stratified associations

Age-stratified analyses showed broadly similar descriptive patterns across both age groups, with lower exercise levels generally associated with higher prevalence of current MDE or GAD (Supplementary Table S1 in S1 File). Among students aged 18–24 years, CMD prevalence declined from 55.4% among those who never or seldom exercised to 33.4% among daily exercisers, with crude RRs showing a graded pattern across intermediate frequency categories. Among students aged 25–35 years, CMD prevalence declined from 56.3% to 41.3%, although the corresponding crude RRs were generally smaller and less clearly graded, with the most pronounced elevation observed in the lowest activity category. Similar but less consistent patterns were observed for exercise intensity, duration, and adherence to the MVPA recommendation, with somewhat stronger gradients among younger students. However, the overall age group × exercise frequency interaction was not statistically significant (Wald χ² = 6.71, df = 4, p = .152). Although one category-specific age interaction term reached statistical significance, the overall interaction test did not support effect modification by age group; the age-stratified findings should therefore be interpreted descriptively.

### Sensitivity analyses

Sensitivity analyses using alternative outcome definitions showed largely consistent findings across both 30-day and 12-month measures of GAD and MDE. For exercise frequency, an inverse dose-response pattern was observed in age-adjusted and sociodemographically adjusted models in both women and men, with lower levels of exercise associated with higher relative risk of anxiety and depression. The strongest associations were seen among those reporting never or seldom exercising, and patterns were similar across time frames. After additional adjustment for baseline psychological distress (Model 3), associations were attenuated but remained in the same direction. In women, modest associations persisted, particularly for GAD, whereas estimates in men were more attenuated and often no longer statistically significant, although point estimates remained elevated. The attenuation following adjustment for HSCL-25 suggests that baseline psychological distress accounts for a substantial proportion of the associations, and these estimates should be interpreted as conservative. Overall, similar patterns were observed for MDE, although associations were generally weaker than for GAD, particularly in fully adjusted models. Detailed results are presented in Supplementary Tables S2 to S5 in S1 File.

In supplementary mutually adjusted models including exercise frequency, intensity, and duration simultaneously, exercise frequency remained the most consistent predictor of CMD, whereas independent associations for duration were weak and inconsistent. Among women, low exercise intensity also remained associated with higher CMD risk.

## Discussion

In this large national cohort of higher-education students, lower levels of physical exercise were prospectively associated with a higher relative risk of clinically defined CMD one year later. Although several associations were attenuated after adjustment for sociodemographic factors and baseline psychological distress, key indicators, particularly exercise frequency and adherence to WHO recommendations, remained robustly associated with subsequent CMD. Associations were most consistent for exercise frequency and adherence to WHO recommendations, whereas intensity and duration showed weaker and sex-specific patterns. Low exercise intensity predicted CMD among women, whereas shorter exercise duration was a stronger risk factor among men. Analyses of total weekly exercise hours further supported a dose-response relationship, with CMD probability declining as exercise volume increased and plateauing at around 10 hours per week. The inverse gradient was somewhat stronger among men, although women had a higher overall prevelence of CMD. Age-stratified analyses showed the same general pattern, although associations were more pronounced and more clearly graded among students aged 18–24 years than among those aged 25–35 years. Taken together, these findings suggest that maintaining regular physical exercise, rather than emphasizing high intensity or long sessions, may be particularly relevant in relation to the likelihood of subsequent CMD.

The present findings add to the existing literature by demonstrating that associations between physical exercise and mental health extend to prospectively assessed, diagnostically defined mental disorders. While numerous studies have linked physical activity to lower levels of depressive and anxiety symptoms, including in student populations [11–14], far fewer have examined clinically defined outcomes using standardized diagnostic instruments. By applying CIDI 5.0 at follow-up, the current study provides evidence that physical exercise is associated with a lower likelihood of meeting DSM-5 criteria for major depressive episode and or generalized anxiety disorder. The graded association observed for exercise frequency and total weekly volume is consistent with findings from large prospective studies and meta-analyses in adult populations showing inverse dose-response relationships between physical activity and incident depression [11–13]. Importantly, these meta-analyses also indicate that the largest relative risk reductions tend to occur when moving from inactivity to moderate activity levels, with weaker incremental benefit at higher volumes. The plateau observed in the present study at higher weekly exercise hours aligns with this pattern and supports the interpretation that accumulated activity may be associated with lower CMD likelihood primarily through regular engagement rather than extreme levels of exertion. Baseline psychological distress (HSCL-25) represents an important consideration. While included as a potential confounder, it may also lie on the pathway between physical exercise and subsequent CMD. Accordingly, models without HSCL-25 adjustment can be interpreted as reflecting total associations, whereas fully adjusted models provide more conservative estimates.

Our finding that exercise frequency was the most consistent correlate of CMD aligns with earlier SHOT studies using symptom-based mental health outcomes, where frequency emerged as a stronger predictor than intensity or duration [16]. Extending this pattern to diagnostically defined CMD suggests that regularity may capture behavioural and psychosocial dimensions of physical activity that are particularly relevant for mental health, such as routine formation, behavioural activation, and sustained engagement. In contrast, the more heterogeneous associations observed for intensity and duration are consistent with previous findings showing that these dimensions vary in importance depending on outcome definition and population characteristics [13].

Sex-specific patterns in the present study should be interpreted in light of earlier evidence indicating that associations between physical activity and mental health may differ between women and men [40]. While these differences were modest, the findings suggest that insufficient intensity may be more relevant for women and insufficient duration for men, potentially reflecting differences in activity preferences, typical training patterns, or baseline risk of mental disorders. However, such interpretations remain tentative and warrant further investigation.

Finally, although age-stratified analyses suggested somewhat stronger and more clearly graded associations among younger students aged 18–24 years, the overall age group × exercise frequency interaction was not statistically significant, indicating that these differences should be interpreted descriptively. The descriptive gradients among younger students may nevertheless be hypothesis-generating. Students in this age group may be more likely to experience behavioural instability during the transition into higher education, including changes in routines, social networks, sleep, and health behaviours. Regular physical exercise may therefore be more closely related to broader patterns of adjustment and daily structure in this group. However, these explanations remain tentative and should be examined in future studies with repeated measures and formal tests of age-related effect modification.

### Implications for prevention and student health promotion

The findings indicate that regular physical exercise may represent a relevant and modifiable correlate of lower CMD likelihood in higher education populations. The consistent associations for exercise frequency and total weekly volume suggest that sustained engagement may be more important than achieving high intensity or long sessions. This supports a focus on regularity and integration of activity into daily routines, rather than performance-oriented targets [12].

Physical activity may be a useful component of broader student mental health promotion efforts. International mental health surveys among college students document both high prevalence of CMD and substantial unmet treatment need among students [2–4]. Although causality cannot be established from the present study, encouraging regular exercise may represent a potentially beneficial component of broader strategies to support student mental health, especially among students with emerging or subthreshold symptoms.

Sustained engagement in physical activity is strongly shaped by contextual and structural factors. Reviews consistently identify lack of time, academic workload, financial constraints, and limited access to suitable facilities as major barriers to regular exercise among university students [41,42]. As a result, strategies relying primarily on individual motivation are unlikely to be sufficient. Institutional approaches that reduce structural barriers, such as accessible facilities, low-threshold programmes, and integration of activity opportunities within academic schedules, may be more likely to support sustained engagement across diverse student groups [43].

Several mechanisms may help explain the observed association between physical exercise and a lower RR of CMD. At the psychological and social level, exercise may be related to self-efficacy, mastery, and perceived control, while also providing structured distraction from ruminative thought patterns [44]. Physical exercise often involves regular routines and social interaction, both of which are independently associated with better mental health and lower CMD likelihood [45]. These processes may help explain why exercise frequency and regularity emerged as the most consistent indicators. Maintaining an active routine, even at moderate intensity, may support resilience to everyday stressors and enhance coping capacity. From a physiological perspective, regular physical exercise may be linked to stress regulation through several pathways, including modulation of the hypothalamic pituitary adrenal axis, reductions in low grade inflammation, improvements in sleep quality, and enhanced neuroplasticity [46]. Together, these psychological, social, and biological mechanisms provide plausible and complementary explanations for the observed association between physical exercise and subsequent CMD.

### Strengths and limitations

The present study has several important strengths. First, it is based on a large, national cohort of higher-education students with prospective follow-up and diagnostic outcomes assessed via the self-administered CIDI 5.0. Second, the use of DSM-5-based diagnostic data provides a clinically relevant endpoint, extending prior SHoT research that has relied primarily on symptom scales. Third, the analytic strategy incorporated inverse probability weighting to address potential bias due to non-response and oversampling, improving population representativeness. Moreover, adjustment for sociodemographic and psychological factors, including baseline psychological distress, provides a stringent test of independent associations. Finally, by incorporating a continuous measure of total weekly exercise hours, in addition to frequency, intensity, duration, and adherence to WHO recommendations, the study offers a more detailed assessment of dose-response relationships between overall activity volume and CMD risk.

However, several limitations should be acknowledged. Physical exercise was self-reported and may be subject to recall and social-desirability bias, which may introduce measurement error and misclassification of exposure. The operationalisation of adherence to WHO recommendations was relatively strict and may have resulted in some misclassification of participants around the threshold. Such misclassification is likely to be largely non-differential with respect to subsequent CMD and would therefore tend to attenuate the observed associations, potentially leading to underestimation of the dose-response gradients. However, some degree of differential misclassification cannot be excluded, particularly if reporting of exercise behaviour is related to underlying mental health. Despite the prospective design, causality cannot be definitively established, and reverse causation remains possible. Further, although the follow-up period of one year provides valuable insight into short-term risk, longer follow-up would be necessary to assess sustained protective effects or bi-directional dynamics over time. Participation bias is another consideration: response rates were modest at baseline, and additional attrition occurred before the CIDI follow-up. Despite the use of weighting, individuals with severe mental-health problems may have been under-represented. IPW was applied to mitigate differential response; however, this approach relies on the assumption that all relevant predictors of participation are measured and correctly specified. Unmeasured factors, such as severity of mental health problems, motivation to participate, or health-related behaviours, may still contribute to residual selection bias. In addition, potential residual confounding from unmeasured factors such as mental health service use, psychotropic medication, and substance use cannot be excluded, as these factors may influence both engagement in physical exercise and the likelihood of CMD. Therefore, although weighting is likely to reduce bias, it cannot fully eliminate it, and the results should be interpreted with this limitation in mind.

The diagnostic data, although based on the CIDI, were collected via web-based self-administration rather than clinical interview, which may affect diagnostic precision compared with clinician-administered tools such as SCID or SCAN. Also, CMD was not assessed using diagnostic criteria at baseline, and participants with prevalent disorders at baseline were therefore not excluded. Consequently, the outcome at follow-up reflects a mixture of incident, persistent, and recurrent cases. The observed associations should therefore be interpreted as relating to subsequent CMD status rather than strictly incident risk. Another limitation relates to the operationalisation of the outcome variable. The use of a composite CMD outcome combining depression and anxiety across different time frames may have obscured disorder-specific and temporal differences, although sensitivity analyses using separate outcomes yielded largely consistent findings. These analyses suggested broadly similar patterns, although associations were somewhat stronger for GAD than for MDE. Finally, the exercise measures were limited to frequency, intensity, duration, and total volume, and did not capture contextual characteristics such as type of activity, social context, enjoyment, or perceived purpose. These factors may be relevant for understanding the association between exercise and mental health and should be considered in future research.

### Conclusions

In summary, lower levels of physical exercise were associated with a higher likelihood of subsequent CMD among students in higher education. Frequent exercise, adherence to weekly activity recommendations, and greater total weekly exercise volume were each linked to reduced CMD risk, with a clear dose-response pattern. Risk declined steeply with increasing activity at lower levels and levelled off at higher volumes, with the lowest risk observed at approximately 5–6 hours per week among women and at progressively higher volumes among men. At these activity levels, CMD likelihood was lower than among inactive students, although the magnitude of the association was substantially attenuated after adjustment for sociodemographic factors and baseline psychological distress. Taken together, these findings underscore the importance of regular and sustained physical exercise as a potential target for student mental health promotion. Promoting consistent and attainable exercise routines, rather than emphasising high intensity or extreme volumes, may represent a pragmatic component of broader student mental health promotion efforts. However, the present findings are observational and should be interpreted as evidence of prospective associations rather than causal effects.

## Supporting information

S1 File Supplementary tables. Contains Supplementary Tables S1-S8, including age-stratified analyses, separate analyses of generalized anxiety disorder and major depressive episode, categorical and continuous analyses of weekly exercise hours, and mutually adjusted analyses of exercise frequency, intensity, and duration.
(DOCX)
