# Supplementary material for: Physical exercise and risk of common mental disorders among higher education students: A national prospective study
Source: PLoS One. 2026 Aug 19;21(8):e0353787. doi: 10.1371/journal.pone.0353787 (PMC13489442; doi:10.1371/journal.pone.0353787)
Supplement: Supplementary file 1 [file pone.0353787.s002.pdf]

Response to Editorial Office

We have now uploaded the full ethics approval documentation for the study.

The project is covered by a Regional Committee for Medical and Health Research Ethics (REK vest, Norway) approval for the SHoT 2022 study, which is a national epidemiological umbrella study. The approval covers the entire study period, including prospective follow-up and registry linkages, with an approved end date of February 7, 2032.

The original approval document (in Norwegian) as well as an English translation have been uploaded. No separate extension documents apply, as the original approval includes the full study period.

We hope this resolves the request.
