# Supplementary material for: Physical exercise and risk of common mental disorders among higher education students: A national prospective study
Source: PLoS One. 2026 Aug 19;21(8):e0353787. doi: 10.1371/journal.pone.0353787 (PMC13489442; doi:10.1371/journal.pone.0353787)
Supplement: Supplementary file 2 [file pone.0353787.s001.docx]

| **Supplementary Table S1.** Prevalence (%) of common mental disorders (CMD) and crude risk ratios (RR) with 95% confidence intervals for current major depressive episode or generalized anxiety disorder, by physical exercise indicators and age group. | | | | |
| --- | --- | --- | --- | --- |
|  | **18-24 years** | | **25-35 years** | |
|  | **CMD %** | **RR (Model 1)** | **CMD %** | **RR (Model 1)** |
| **Exercise frequency** |  |  |  |  |
| Never/seldom | 55.4 | 1.66 (1.48-1.86) | 56.3 | 1.36 (1.21-1.54) |
| 1 time/week | 47.9 | 1.43 (1.27-1.62) | 49.9 | 1.21 (1.06-1.38) |
| 2-3 times/week | 40.6 | 1.22 (1.09-1.35) | 45.1 | 1.09 (0.98-1.22) |
| 4-5 times/week | 38.7 | 1.16 (1.03-1.30) | 44.8 | 1.09 (0.96-1.23) |
| Every day (ref) | 33.4 | 1.00 | 41.3 | 1.00 |
| **Exercise intensity (average)** |  |  |  |  |
| Easy (no sweat/out of breath) | 51.1 | 1.59 (1.36-1.86) | 55.1 | 1.39 (1.18-1.64) |
| Hard (sweat/out of breath) | 38.3 | 1.19 (1.02-1.39) | 43.2 | 1.09 (0.93-1.28) |
| Almost to exhaustion (ref) | 32.1 | 1.00 | 39.5 | 1.00 |
| **Exercise duration (average)** |  |  |  |  |
| <15 minutes | 56.3 | 1.58 (1.29-1.94) | 52.2 | 1.16 (0.91-1.47) |
| 15-29 minutes | 50.8 | 1.43 (1.29-1.58) | 54.3 | 1.20 (1.08-1.34) |
| 30-60 minutes | 42.3 | 1.19 (1.10-1.28) | 44.2 | 0.98 (0.90-1.06) |
| >1 hour (ref) | 35.6 | 1.00 | 45.2 | 1.00 |
| **Meets MVPA ≥150 min/week** |  |  |  |  |
| Below recommendation | 45.7 | 1.36 (1.27-1.47) | 49.3 | 1.26 (1.16-1.38) |
| Above recommendation | 33.5 | 1.00 | 39.1 | 1.00 |
|  | | | | |

| **Supplementary Table S2.** Estimated prevalence and relative risk (RR) of 30-day generalized anxiety disorder (GAD) by exercise frequency, stratified by sex. | | | | |
| --- | --- | --- | --- | --- |
| **FEMALES** | **GAD 30-day %** | **RR (Model 1)** | **RR (Model 2)** | **RR (Model 3)** |
| **Exercise frequency** |  |  |  |  |
| Never/seldom | 21.0% | 1.74 (1.42–2.13) | 1.54 (1.19–2.00) | 1.18 (0.93–1.51) |
| 1 time/week | 18.7% | 1.54 (1.25–1.91) | 1.55 (1.20–2.00) | 1.32 (1.05–1.67) |
| 2-3 times/week | 15.8% | 1.31 (1.09–1.57) | 1.33 (1.08–1.65) | 1.22 (1.00–1.49) |
| 4-5 times/week | 13.5% | 1.12 (0.92–1.36) | 1.20 (0.95–1.51) | 1.15 (0.93–1.43) |
| Every day (ref) | 12.0% | 1.00 | 1.00 | 1.00 |
|  |  |  |  |  |
| **MALES** |  |  |  |  |
| **Exercise frequency** | **GAD 30-day %** | **RR (Model 1)** | **RR (Model 2)** | **RR (Model 3)** |
| Never/seldom | 12.6% | 2.57 (1.61–4.09) | 2.16 (1.22–3.80) | 1.21 (0.70–2.09) |
| 1 time/week | 10.0% | 2.13 (1.29–3.52) | 2.37 (1.33–4.23) | 1.52 (0.89–2.62) |
| 2-3 times/week | 6.9% | 1.44 (0.91–2.26) | 1.41 (0.83–2.41) | 0.93 (0.56–1.56) |
| 4-5 times/week | 7.9% | 1.70 (1.07–2.73) | 1.57 (0.90–2.73) | 1.15 (0.67–1.95) |
| Every day (ref) | 4.8% | 1.00 | 1.00 | 1.00 |
| Note: Model 1: adjusted for age. Model 2: additionally adjusted for sociodemographic covariates (civil status, income, immigrant background, BMI). Model 3: additionally adjusted for baseline psychological distress (HSCL-25). Estimates are weighted using inverse probability weights. | | | | |

| **Supplementary Table S3.** Estimated prevalence and relative risk (RR) of 12-month generalized anxiety disorder (GAD). | | | | |
| --- | --- | --- | --- | --- |
| **FEMALES** | **GAD 12-month %** | **RR (Model 1)** | **RR (Model 2)** | **RR (Model 3)** |
| **Exercise frequency** |  |  |  |  |
| Never/seldom | 30.4% | 1.56 (1.33–1.84) | 1.44 (1.18–1.75) | 1.14 (0.95–1.37) |
| 1 time/week | 27.0% | 1.39 (1.17–1.65) | 1.32 (1.08–1.60) | 1.15 (0.96–1.37) |
| 2-3 times/week | 22.6% | 1.17 (1.01–1.34) | 1.13 (0.96–1.33) | 1.06 (0.91–1.23) |
| 4-5 times/week | 21.3% | 1.10 (0.94–1.29) | 1.12 (0.94–1.33) | 1.08 (0.92–1.27) |
| Every day (ref) | 19.3% | 1.00 | 1.00 | 1.00 |
|  |  |  |  |  |
| **MALES** |  |  |  |  |
| **Exercise frequency** | **GAD 12-month %** | **RR (Model 1)** | **RR (Model 2)** | **RR (Model 3)** |
| Never/seldom | 19.7% | 2.35 (1.64–3.35) | 2.13 (1.37–3.31) | 1.30 (0.86–1.96) |
| 1 time/week | 16.6% | 2.07 (1.41–3.03) | 2.46 (1.58–3.83) | 1.66 (1.10–2.52) |
| 2-3 times/week | 10.8% | 1.33 (0.94–1.89) | 1.28 (0.84–1.94) | 0.92 (0.62–1.36) |
| 4-5 times/week | 12.6% | 1.58 (1.10–2.27) | 1.61 (1.05–2.45) | 1.22 (0.82–1.82) |
| Every day (ref) | 8.1% | 1.00 | 1.00 | 1.00 |
| Note: Model 1: adjusted for age. Model 2: additionally adjusted for sociodemographic covariates (civil status, income, immigrant background, BMI). Model 3: additionally adjusted for baseline psychological distress (HSCL-25). Estimates are weighted using inverse probability weights. | | | | |

| **Supplementary Table S4.** Estimated prevalence and relative risk (RR) of 30-day major depressive episode (MDE) | | | | |
| --- | --- | --- | --- | --- |
| **FEMALES** | **MDE 30-day %** | **RR (Model 1)** | **RR (Model 2)** | **RR (Model 3)** |
| **Exercise frequency** |  |  |  |  |
| Never/seldom | 24.6% | 1.72 (1.44–2.07) | 1.53 (1.21–1.92) | 1.14 (0.92–1.41) |
| 1 time/week | 18.5% | 1.30 (1.06–1.59) | 1.15 (0.89–1.47) | 0.96 (0.76–1.21) |
| 2-3 times/week | 16.2% | 1.13 (0.96–1.34) | 1.13 (0.93–1.38) | 1.03 (0.86–1.23) |
| 4-5 times/week | 13.5% | 0.95 (0.79–1.15) | 0.97 (0.78–1.20) | 0.93 (0.76–1.13) |
| Every day (ref) | 14.2% | 1.00 | 1.00 | 1.00 |
|  |  |  |  |  |
| **MALES** |  |  |  |  |
| **Exercise frequency** | **MDE 30-day %** | **RR (Model 1)** | **RR (Model 2)** | **RR (Model 3)** |
| Never/seldom | 18.6% | 2.67 (1.83–3.89) | 2.41 (1.52–3.82) | 1.37 (0.90–2.09) |
| 1 time/week | 12.5% | 1.82 (1.19–2.77) | 2.13 (1.31–3.46) | 1.37 (0.87–2.15) |
| 2-3 times/week | 8.6% | 1.26 (0.86–1.84) | 1.19 (0.76–1.86) | 0.79 (0.52–1.21) |
| 4-5 times/week | 7.9% | 1.18 (0.78–1.79) | 1.10 (0.68–1.79) | 0.81 (0.52–1.28) |
| Every day (ref) | 6.9% | 1.00 | 1.00 | 1.00 |
| Note: Model 1: adjusted for age. Model 2: additionally adjusted for sociodemographic covariates (civil status, income, immigrant background, BMI). Model 3: additionally adjusted for baseline psychological distress (HSCL-25). Estimates are weighted using inverse probability weights. | | | | |

| **Supplementary Table S5.** Estimated prevalence and relative risk (RR) of 12-month major depressive episode (MDE). | | | | |
| --- | --- | --- | --- | --- |
| **FEMALES** | **MDE 12-month %** | **RR (Model 1)** | **RR (Model 2)** | **RR (Model 3)** |
| **Exercise frequency** |  |  |  |  |
| Never/seldom | 43.9% | 1.45 (1.29–1.63) | 1.38 (1.20–1.60) | 1.14 (1.00–1.30) |
| 1 time/week | 38.2% | 1.27 (1.12–1.43) | 1.23 (1.06–1.43) | 1.09 (0.95–1.25) |
| 2-3 times/week | 33.9% | 1.13 (1.02–1.25) | 1.16 (1.02–1.30) | 1.09 (0.98–1.22) |
| 4-5 times/week | 32.1% | 1.07 (0.95–1.19) | 1.11 (0.97–1.26) | 1.08 (0.96–1.21) |
| Every day (ref) | 30.1% | 1.00 | 1.00 | 1.00 |
|  |  |  |  |  |
| **MALES** |  |  |  |  |
| **Exercise frequency** | **MDE 12-month %** | **RR (Model 1)** | **RR (Model 2)** | **RR (Model 3)** |
| Never/seldom | 36.2% | 1.97 (1.57–2.46) | 1.86 (1.41–2.44) | 1.29 (1.00–1.67) |
| 1 time/week | 27.1% | 1.50 (1.16–1.92) | 1.56 (1.16–2.09) | 1.17 (0.89–1.55) |
| 2-3 times/week | 22.9% | 1.26 (1.02–1.57) | 1.23 (0.96–1.59) | 0.97 (0.77–1.24) |
| 4-5 times/week | 20.4% | 1.13 (0.89–1.44) | 1.13 (0.85–1.49) | 0.94 (0.72–1.21) |
| Every day (ref) | 18.3% | 1.00 | 1.00 | 1.00 |
| Note: Model 1: adjusted for age. Model 2: additionally adjusted for sociodemographic covariates (civil status, income, immigrant background, BMI). Model 3: additionally adjusted for baseline psychological distress (HSCL-25). Estimates are weighted using inverse probability weights. | | | | |

| **Supplementary Table S6.** Estimated prevalence and relative risk (RR) of common mental disorder (CMD) by weekly exercise hours, stratified by sex | | | | |
| --- | --- | --- | --- | --- |
| **FEMALES** |  |  |  |  |
| **Weekly exercise hours** | **CMD %** | **RR (Model A)** | **RR (Model B)** |  |
| 0 h | 59.9% | 1.25 (1.06-1.48) | 1.07 (0.92-1.24) |  |
| 1-2 h | 50.3% | 1.06 (0.92-1.23) | 0.97 (0.85-1.11) |  |
| 3-4 h | 46.0% | 0.99 (0.86-1.15) | 0.97 (0.85-1.10) |  |
| 5-6 h | 41.5% | 0.91 (0.78-1.06) | 0.89 (0.78-1.02) |  |
| 7-9 h | 42.9% | 0.98 (0.83-1.16) | 0.94 (0.81-1.09) |  |
| 10+ h (ref) | 44.9% | 1.00 | 1.00 |  |
| **MALES** |  |  |  |  |
| **Weekly exercise hours** | **CMD %** | **RR (Model A)** | **RR (Model B)** |  |
| 0 h | 51.5% | 2.71 (1.94-3.78) | 1.96 (1.44-2.67) |  |
| 1-2 h | 34.3% | 1.85 (1.36-2.52) | 1.41 (1.06-1.86) |  |
| 3-4 h | 29.2% | 1.53 (1.12-2.10) | 1.25 (0.94-1.66) |  |
| 5-6 h | 29.6% | 1.50 (1.08-2.08) | 1.28 (0.95-1.71) |  |
| 7-9 h | 23.8% | 1.30 (0.90-1.86) | 1.25 (0.90-1.74) |  |
| 10+ h (ref) | 19.6% | 1.00 | 1.00 |  |
| Note: Model A adjusted for age, civil status, income, immigrant background, and BMI category. Model B additionally adjusted for baseline psychological distress (HSCL-25). Weekly exercise hours were categorised as 0, 1-2, 3-4, 5-6, 7-9, and 10+ hours per week. Estimates are weighted using inverse probability weights. | | | | |

| **Supplementary Table S7.** Mutually adjusted associations between exercise frequency, intensity, duration, and common mental disorder (CMD), stratified by sex | | | |
| --- | --- | --- | --- |
| **FEMALES** |  |  |  |
| **Exercise indicator** | **Category** | **RR (Model A)** | **RR (Model B)** |
| **Exercise frequency** | Never/seldom | 1.25 (1.09-1.43) | 1.09 (0.97-1.23) |
|  | 1 time/week | 1.17 (1.04-1.33) | 1.06 (0.95-1.18) |
|  | 2-3 times/week | 1.13 (1.02-1.24) | 1.06 (0.97-1.16) |
|  | 4-5 times/week | 1.14 (1.03-1.27) | 1.10 (1.00-1.20) |
|  | Every day (ref) | 1.00 | 1.00 |
| **Exercise intensity** | Easy | 1.35 (1.13-1.61) | 1.31 (1.12-1.52) |
|  | Hard | 1.12 (0.94-1.32) | 1.16 (1.00-1.35) |
|  | Almost exhausted (ref) | 1.00 | 1.00 |
| **Exercise duration** | <15 minutes | 1.05 (0.82-1.33) | 1.07 (0.88-1.29) |
|  | 15-29 minutes | 1.04 (0.93-1.15) | 0.99 (0.90-1.08) |
|  | 30-60 minutes | 0.94 (0.87-1.01) | 0.96 (0.90-1.02) |
|  | >1 hour (ref) | 1.00 | 1.00 |
| **MALES** |  |  |  |
| **Exercise indicator** | **Category** | **RR (Model A)** | **RR (Model B)** |
| **Exercise frequency** | Never/seldom | 1.72 (1.32-2.26) | 1.37 (1.08-1.75) |
|  | 1 time/week | 1.61 (1.23-2.10) | 1.25 (0.98-1.60) |
|  | 2-3 times/week | 1.20 (0.95-1.51) | 0.97 (0.78-1.20) |
|  | 4-5 times/week | 1.24 (0.97-1.58) | 1.02 (0.81-1.27) |
|  | Every day (ref) | 1.00 | 1.00 |
| **Exercise intensity** | Easy | 1.01 (0.78-1.32) | 1.04 (0.83-1.30) |
|  | Hard | 0.90 (0.72-1.13) | 0.99 (0.82-1.20) |
|  | Almost exhausted (ref) | 1.00 | 1.00 |
| **Exercise duration** | <15 minutes | 1.22 (0.77-1.91) | 1.30 (0.88-1.92) |
|  | 15-29 minutes | 1.29 (1.01-1.63) | 1.10 (0.89-1.36) |
|  | 30-60 minutes | 1.14 (0.96-1.34) | 1.07 (0.92-1.23) |
|  | >1 hour (ref) | 1.00 | 1.00 |
| Note: Model A adjusted for age, civil status, income, immigrant background, and BMI category. Model B additionally adjusted for baseline psychological distress (HSCL-25). Frequency, intensity, and duration were entered simultaneously in the same model. Estimates are weighted using inverse probability weights and restricted to participants with complete data on all exercise indicators. | | | |

| **Supplementary Table S8.** Continuous association between weekly exercise hours and CMD (linear and quadratic terms) | | | | |
| --- | --- | --- | --- | --- |
| **FEMALES** |  |  |  |  |
| **Variable** | **RR** | **95% CI** | **p-value** |  |
| Weekly hours (linear) | 0.964 | 0.939 to 0.990 | 0.007 |  |
| Weekly hours² (quadratic) | 1.003 | 1.000 to 1.005 | 0.024 |  |
| **MALES** |  |  |  |  |
| **Variable** | **RR** | **95% CI** | **p-value** |  |
| Weekly hours (linear) | 0.931 | 0.879 to 0.986 | 0.014 |  |
| Weekly hours² (quadratic) | 1.003 | 0.998 to 1.007 | 0.288 |  |
| Note: Poisson regression with robust variance. Adjusted for age, civil status, income, immigrant background, BMI category, and HSCL-25. | | | | |
