# Supplementary material for: Physical exercise and risk of common mental disorders among higher education students: A national prospective study
Source: PLoS One. 2026 Aug 19;21(8):e0353787. doi: 10.1371/journal.pone.0353787 (PMC13489442; doi:10.1371/journal.pone.0353787)
Supplement: Supplementary file 3 [file pone.0353787.s003.docx]

Dear Editor,

We thank you and the reviewers for the careful and constructive evaluation of our manuscript, “Physical exercise and subsequent common mental disorders among higher education students: A national prospective study.” We have revised the manuscript thoroughly in response to the comments and believe that the changes have substantially strengthened the clarity, methodological transparency, and overall contribution of the study.

In particular, we have clarified the study’s motivation and contribution, expanded the literature review to include recent high-quality studies, improved the definition and interpretation of the outcome, and conducted additional analyses to address concerns regarding the operationalisation of exercise variables and outcome heterogeneity. We have also strengthened the discussion of limitations, including selection bias, residual confounding, and the role of baseline psychological distress, and revised the manuscript throughout to avoid causal language and improve conceptual precision. Below, we provide a detailed, point-by-point response to all reviewer comments. All changes have been incorporated into the revised manuscript.

Reviewer #1: Review Comments
This study uses data from a national survey of university students in Norway to investigate the association between physical exercise and common mental disorders (CMD), defined as depression and anxiety disorders based on clinical criteria. As expected, the authors report associations between exercise indicators and CMD, and they also report differential effects by sex based on stratified analyses.
Overall, I recommend major revision before the manuscript can be considered for publication. I hope the authors can address the following concerns.
1. Motivation and contribution
The motivation of the study is not sufficiently developed. The authors emphasize that the outcome is based on clinically defined common mental disorders rather than symptom scales. However, it is not clear how this outcome substantially differs from commonly used mental health scales in terms of the implications for the research question. The association between physical exercise and mental health outcomes is already well established in the literature. Therefore, the manuscript should more clearly articulate how the present study advances the substantive literature. Even if PLOS ONE does not require highly novel findings, the authors should better justify the added value of this work.

- Response: We thank the reviewer for this helpful comment. We have revised the Introduction to more clearly articulate the motivation and contribution of the study. In particular, we now clarify that the use of diagnostically defined outcomes (CIDI 5.0) represents an important extension beyond symptom-based measures, as it allows examination of associations with clinically relevant disorders rather than levels of psychological distress. While prior research has consistently documented associations between physical exercise and mental health symptoms, fewer studies have investigated these relationships prospectively using standardized diagnostic criteria.
  We have also strengthened the framing of the study’s contribution by emphasizing the combined use of a large national cohort, prospective design, multiple dimensions of exercise (including total weekly volume), and analytic strategies addressing selection bias and baseline distress. Together, these features strengthen the clinical relevance and methodological robustness of the study.

2. Construction of the outcome variable
The definition of the outcome variable raises several concerns. If I understand correctly, CMD is defined as the presence of either major depressive episode (MDE) or generalized anxiety disorder (GAD) based on CIDI diagnostic criteria. In addition, diagnoses from two time frames (past 30 days or past 12 months) appear to be combined into a single binary outcome. Thus, multiple diagnostic definitions (MDE vs. GAD and 30-day vs. 12-month diagnoses) are effectively collapsed into a single CMD indicator using an OR rule.
This approach raises several conceptual and interpretational issues.
First, although diagnostic classifications are often treated as binary outcomes in epidemiological research, this approach inevitably reduces information about symptom severity and heterogeneity within disorders. While this may be acceptable depending on the research question, the potential loss of information should at least be acknowledged.
Second, combining two distinct disorders (depression and anxiety) and two temporal diagnostic definitions (30-day and 12-month) into a single outcome may obscure meaningful differences. These represent different clinical constructs and different time frames of disorder occurrence. As currently specified, it becomes difficult to determine whether the observed associations reflect depression, anxiety, recent episodes, or more distal 12-month diagnoses.
I would therefore recommend presenting additional analyses separating the diagnostic components (e.g., MDE vs. GAD, and possibly 30-day vs. 12-month diagnoses) to better clarify the pattern of associations. Such analyses would help readers understand whether the reported results are driven primarily by one disorder or one diagnostic time frame.

- Response: We thank the reviewer for this constructive comment. We agree that combining MDE and GAD, as well as 30-day and 12-month diagnoses, into a single CMD outcome may obscure potentially meaningful differences across disorders and time frames.

The primary rationale for using a composite CMD outcome was to capture overall burden of clinically defined common mental disorders, in line with previous epidemiological research, and to maximise statistical power. However, we acknowledge that this approach reduces specificity and may mask heterogeneity in associations. In response to the reviewer’s suggestion, we have now conducted additional sensitivity analyses separating the diagnostic components. Specifically, we estimated models for 30-day and 12-month GAD and MDE as separate outcomes. These analyses were performed using the same modelling framework and adjustment strategy as the main analyses.
The results from these sensitivity analyses showed largely consistent patterns across outcomes, with similar inverse dose-response associations between physical exercise frequency and both GAD and MDE. Associations were generally somewhat stronger for GAD than for MDE, but the overall pattern was robust across both diagnostic categories and time frames. As in the main analyses, estimates were attenuated after adjustment for baseline psychological distress.
These additional results are now presented in Supplementary Tables S2 to S5 and are briefly described in the Results section. We have also expanded the Discussion to acknowledge the limitations of the composite CMD outcome, including potential loss of information and heterogeneity across disorders and time frames.

3. Treatment of exercise variables
The exercise variables are analyzed individually without controlling for other exercise-related variables. This approach makes interpretation difficult because different indicators of exercise (e.g., frequency, intensity, duration) are likely correlated.
For example, the analysis of meetin g the MVPA criterion (>150 minutes/week) essentially compares individuals below and above the guideline threshold. However, this does not address whether meaningful differences exist around that threshold (e.g., between 120–150 minutes and 150–180 minutes per week).
A more informative analysis would examine:
• finer categories of weekly exercise hours and compare adjacent categories, or
• a continuous specification of exercise hours with a nonlinear or curvilinear term, allowing examination of the instantaneous rate of change around the guideline threshold.
Furthermore, the models examining frequency, intensity, and duration do not appear to control for each other. Because these variables are related, it is not surprising that they all show associations with the outcome. Joint models including multiple exercise indicators would provide more interpretable results.

- Response: We thank the reviewer for this helpful and constructive comment.

To address the concern regarding the treatment of exercise variables, we conducted additional analyses of weekly exercise hours using both categorical and continuous specifications. Specifically, we modelled weekly exercise hours in finer categories and, in separate models, as a continuous exposure including both linear and quadratic terms to formally assess potential non-linearity. These analyses showed an inverse association between weekly exercise hours and CMD in both sexes. Among women, the quadratic term indicated a curvilinear association with diminishing marginal benefits at higher activity levels, whereas among men the association was more nearly linear.

In response to the suggestion regarding correlated exercise indicators, we also estimated supplementary mutually adjusted models including exercise frequency, intensity, and duration simultaneously. In these models, exercise frequency remained the most consistent predictor of CMD, whereas independent associations for duration were weak and inconsistent. Among women, lower exercise intensity remained associated with higher relative risk of CMD. In the main analyses, frequency, intensity, and duration were examined separately to provide a clear and interpretable description of their individual associations with CMD, which was the primary aim of the study. However, the supplementary mutually adjusted models suggest that exercise frequency captures much of the relevant variation in exercise behaviour in relation to CMD.

Finally, we note that the mutually adjusted models were based on a reduced subsample, as intensity and duration were only assessed among participants reporting regular exercise. These results are therefore presented as supplementary and interpreted with caution.

We have now clarified these additional analyses in the manuscript and present the corresponding results in the Supplementary Material.

4. Missing descriptive statistics
The manuscript does not provide a descriptive statistics table for the exercise variables. This omission is problematic because readers cannot understand the distribution of the key independent variables. At minimum, the manuscript should present summary statistics (means, standard deviations, and/or category frequencies) for all exercise measures.

- Response: We agree that presenting descriptive statistics for the key exposure variables is essential for interpreting the findings. In response, we have added a new table (Table 2) showing the distribution of all physical exercise indicators, including frequency, intensity, duration, and weekly exercise hours, stratified by sex. The table presents category-specific counts and percentages based on available cases for each variable. Exercise intensity and duration are reported among participants with regular exercise, as these items were only assessed in this subgroup.

5. Lack of exercise measures at follow-up
Exercise variables were measured only at Time 1, while CMD was measured at Time 2 approximately one year later. Without measuring exercise at Time 2, it is difficult to evaluate changes in behavior over time.
More importantly, the manuscript occasionally uses causal language (e.g., “reduce” or “protective factor”). Given the observational design and the absence of repeated measures of both exposure and outcome, such causal interpretations are not justified. In longitudinal research, stronger causal claims are typically supported by designs such as cross-lagged panel models with repeated measures of both constructs. At minimum, the authors should avoid causal language and interpret the findings more cautiously.

- Response: We thank the reviewer for this important comment. We acknowledge that physical exercise was measured only at baseline, while CMD outcomes were assessed at follow-up, and that we were therefore unable to examine changes in exercise behavior over time. This is a limitation of the study, and we have now clarified this more explicitly in the Discussion section.
  We also agree that causal interpretations are not warranted given the observational design and the absence of repeated measures of both exposure and outcome. In response, we have carefully revised the manuscript to avoid causal language such as “reduce” and “protective factor,” and instead use more appropriate terms such as “associated with” or “linked to.” We have also emphasized that the findings should be interpreted as prospective associations rather than evidence of causal effects.

6. Justification of the one-year interval
The manuscript does not sufficiently justify the one-year interval between baseline and follow-up. The authors should explain why this time lag is theoretically or clinically meaningful for studying the relationship between exercise and the onset of CMD.

- Response: We agree that the rationale for the follow-up interval should be clarified. The one-year interval in the present study was not specifically chosen to examine the relationship between exercise and CMD, but reflects the design of the SHoT follow-up study, which was conducted to assess diagnostic outcomes using a standardized CIDI-based interview and to estimate the prevalence of common mental disorders.
  Within this framework, the approximately one-year interval provides a reasonable temporal separation between baseline exposures and subsequent mental health outcomes, allowing for the assessment of prospective associations. At the same time, we acknowledge that this interval may not be optimal for capturing the dynamic nature of exercise behaviour or for identifying the most relevant exposure window. Shorter intervals could better capture changes in behaviour, while longer intervals might be more informative for longer-term risk patterns.
  We have now clarified this in the manuscript and explicitly acknowledge that the study design limits the ability to examine changes in exercise over time and to draw conclusions about the most relevant temporal relationship between exercise and CMD.

7. Literature review
The literature review is somewhat limited. A broader and more up-to-date review of the literature on physical exercise and mental health is needed. For example, a quick search reveals relevant studies such as Chiang et al. (2024) in JAMA Pediatrics that are not discussed. The manuscript would benefit from a more comprehensive review of recent work in this area.

- Response: We thank the reviewer for this important suggestion. We have expanded the literature review to incorporate recent high-quality studies, including large cohort and meta-analytic evidence published after 2022. Specifically, we now cite Chiang et al. (2024), a nationwide cohort study in JAMA Pediatrics showing that higher physical fitness in childhood and adolescence was associated with lower subsequent risk of mental disorders. We also added recent student-population and meta-analytic evidence on physical activity and mental health, including studies of university students and updated systematic reviews.

8. Inconsistent statistical models
The main analyses use modified Poisson regression to estimate risk ratios, whereas the supplementary analyses for weekly exercise hours rely on logistic regression with conversion of predicted probabilities to relative risks. The authors should clarify the rationale for using different modeling approaches across analyses. It would also be helpful to report whether similar results are obtained if the continuous exposure analyses are estimated using the same modified Poisson framework as the primary models.

- Response: We thank the reviewer for this important comment. We agree that consistency in the modelling framework is preferable. In response, we have re-estimated the analyses of weekly exercise hours using the same modified Poisson regression approach with robust standard errors as applied in the main analyses, rather than logistic regression. Weekly exercise hours were modelled as a continuous exposure including both linear and quadratic terms to assess potential non-linearity, and predicted relative risks were derived from these models using 0 hours per week as the reference category.
  The results obtained using the modified Poisson framework were substantively similar to those from the original logistic models, with no meaningful changes in the overall pattern or interpretation of the associations. The figure has been updated accordingly and is now based on the modified Poisson models, ensuring consistency across all analyses presented in the manuscript.

9. Testing sex and age differences
The manuscript reports separate analyses for men and women and for different age groups. However, stratified analyses alone do not formally test whether the associations differ across groups. A more standard approach would include interaction terms (e.g., sex × exercise variables or age group × exercise variables) to test whether the slopes differ significantly between groups.

- Response: We thank the reviewer for this important comment. We agree that stratified analyses alone do not formally test whether associations differ across groups. In response, we estimated interaction models within the same modified Poisson framework used in the main analyses, including sex × exercise frequency and age group × exercise frequency interaction terms.

These analyses provided evidence that the association between exercise frequency and CMD differed by sex, particularly at the lowest exercise frequency levels. In contrast, evidence for interaction by age group was weaker and less consistent, with only limited support for meaningful differences across age strata. We have now clarified this in the revised manuscript and interpret the stratified analyses accordingly.

10. Use of Python
The methods section states that analyses were performed in SPSS and Python. The role of Python in the analysis is not explained. The authors should clarify which analyses were conducted in Python and why this additional software was used.

- Response: We apologize for the lack of clarity in the original manuscript. Python was initially used for supplementary data handling and visualization; however, in the revised analyses all statistical models and figures have been re-estimated and produced using R. The methods section has been updated accordingly, and references to Python have been removed to avoid confusion.

11. Type of exercise
The manuscript focuses primarily on the amount of exercise (e.g., frequency, duration, and total weekly hours). It would also be informative to consider different types of exercise (for example, social vs. individual activities), as these may have different associations with mental health outcomes. I recognize that such information may not be available in the current dataset and therefore may not be addressable in the present study. Nevertheless, incorporating distinctions in exercise type in future research could provide a more nuanced understanding of the relationship between physical activity and mental health.

- Response: We thank the reviewer for this thoughtful suggestion. We agree that different types of exercise, such as social versus individual activities, may have distinct associations with mental health outcomes and could provide a more nuanced understanding of the relationship. Unfortunately, such information was not available in the SHOT dataset and could therefore not be examined in the present study. We have now acknowledged this as a limitation in the Discussion and highlighted the importance of incorporating more detailed measures of exercise type in future research.

12. Editorial and submission issues
Several elements required for review appear to be missing, including page numbers, tables, and supplementary materials. This made it difficult to identify specific locations in the manuscript when preparing this review. Please ensure that all materials are included in future submissions.

- Response: We thank the reviewer for noting this issue and apologize for the inconvenience caused. In the revised submission, we have ensured that all required elements are included and clearly presented, including page numbers, tables, figures, and supplementary materials. We have also carefully checked that all tables and supplementary files are properly labelled and referenced in the manuscript to facilitate review.

13. Terminology consistency
The manuscript appears to use the terms prevalence and risk ratio somewhat interchangeably in the Results section. These concepts refer to different quantities and should be distinguished more clearly.
Specifically, prevalence refers to the proportion of participants with CMD at follow-up, whereas the regression models estimate risk ratios, which represent the relative probability of CMD between exposure groups. When describing descriptive statistics, it would be more appropriate to refer to prevalence. When interpreting the regression models, the text should consistently refer to risk ratios (or relative risks).
Clarifying this distinction would improve conceptual precision and avoid potential confusion for readers.

- Response: We thank the reviewer for this important clarification. We agree that prevalence and risk ratios represent distinct concepts and should be described consistently. In the revised manuscript, we have carefully distinguished between these terms throughout the Results section. Descriptive estimates are now consistently referred to as prevalence (i.e., the proportion of participants with CMD at follow-up), whereas results from regression models are described using risk ratios (RR), reflecting relative differences between exposure groups. We have revised the wording accordingly to improve conceptual precision and clarity for the reader.

14. Interpretation of adjusted vs. unadjusted estimates
In the Results section (“Association between weekly exercise and CMD”), the authors state that “adjusted and unadjusted estimates were similar, suggesting limited confounding.” However, the curves in the figure appear different. The authors should clarify this interpretation and explain how similarity between adjusted and unadjusted estimates was assessed.

- Response: We thank the reviewer for this important observation. We agree that the original wording overstated the similarity between adjusted and unadjusted estimates. As shown in Figure 2, adjustment leads to noticeable attenuation of the associations, particularly among women at higher levels of weekly exercise, where the adjusted curve is less steep and shows a clearer plateau compared with the unadjusted curve.
  However, the overall pattern of association remains consistent across models, with both adjusted and unadjusted estimates showing an inverse relationship between weekly exercise hours and CMD, and evidence of diminishing returns at higher activity levels. We have revised the text to clarify that similarity refers to the general shape and direction of the association rather than identical effect sizes. We now describe the results as showing attenuation after adjustment rather than limited confounding.

 Reviewer #2: General Comments

This manuscript presents a large, national prospective cohort study examining the association between multiple dimensions of physical exercise and subsequent common mental disorders (CMD) among higher education students in Norway. The study addresses an important public health question and contributes meaningfully to the literature by utilising diagnostically defined outcomes (CIDI 5.0) rather than symptom-based measures.

Overall, the manuscript is well-written, methodologically rigorous, and highly relevant. The use of inverse probability weighting, stratified analyses, and multiple operationalisations of physical exercise are notable strengths. The findings are consistent with existing evidence and provide additional insight into dose-response relationships and the relative importance of exercise frequency.

However, several important methodological and interpretational issues require clarification, particularly regarding selection bias, causal interpretation, and the role of baseline psychological distress in the analytical framework.

Major Comments
1. Selection Bias and Representativeness

The analytic sample represents a relatively small proportion of the baseline cohort (10,460 of 53,362; ~17.6%). Although inverse probability weighting (IPW) was applied to address differential response, this approach relies on the assumption that all relevant predictors of non-response are measured and correctly specified.
The manuscript acknowledges minimal differences in observed variables; however, unmeasured factors (e.g., severity of mental illness, motivation, health behaviours) may still bias the results. The discussion would benefit from a more explicit acknowledgement of the limitations of IPW and the potential for residual bias arising from unmeasured factors. Where possible, further justification for the representativeness of the weighted sample would strengthen confidence in the findings.

- Response: We thank the reviewer for this important comment. We agree that the relatively low participation rate in the CIDI follow-up and the reliance on inverse probability weighting (IPW) warrant careful consideration.
  In the revised manuscript, we have clarified the assumptions underlying the IPW approach, including that it relies on correctly specified models and measured predictors of response. While we included a range of sociodemographic variables and baseline psychological distress (HSCL-25) in the weighting model, we acknowledge that unmeasured factors, such as severity of mental illness, motivation, or health-related behaviours, may still contribute to residual selection bias.
  To address this, we have expanded the Discussion to more explicitly acknowledge the potential for residual bias despite weighting. At the same time, we note that observed differences between responders and non-responders were small, and that IPW-adjusted estimates are therefore likely to reduce, although not eliminate, bias due to differential participation. These clarifications have been added to improve transparency and interpretation of the findings.

2. Adjustment for Baseline Psychological Distress (HSCL-25)

The inclusion of baseline psychological distress (HSCL-25) in the fully adjusted model is an important but conceptually complex decision. HSCL-25 may act as a confounder but may also lie on the causal pathway between physical exercise and CMD. Adjusting for it may therefore attenuate the total effect and instead estimate a direct effect.

The manuscript would benefit from clearer articulation of the conceptual role of HSCL-25 within the analytical framework. Distinguishing between confounding and mediation, and interpreting Models 2 and 3 accordingly, would improve clarity. Explicit reference to an underlying causal framework (e.g., a directed acyclic graph) would further strengthen the justification.

- Response: We thank the reviewer for this thoughtful and important comment. We agree that baseline psychological distress (HSCL-25) may play a dual role as both a confounder and a potential mediator in the association between physical exercise and subsequent CMD.
  In the revised manuscript, we have clarified this conceptual distinction. Specifically, we now interpret Model 2 (adjusted for sociodemographic factors) as approximating the total association between exercise and CMD, whereas Model 3 (additionally adjusted for HSCL-25) is interpreted as a more conservative estimate that accounts for baseline psychological distress, and may be viewed as approximating a direct association not operating through baseline distress.
  We also acknowledge that adjusting for HSCL-25 may lead to attenuation of estimates if psychological distress lies on the pathway between exercise and later CMD. This has now been explicitly stated in both the Methods and Discussion sections. While we did not formally specify a directed acyclic graph, the revised text now makes the underlying causal assumptions more transparent.

3. Outcome Definition and Temporal Interpretation
CMD is defined as 30-day or 12-month MDE/GAD at follow-up. It is unclear whether participants with CMD at baseline were excluded from the analyses. If baseline cases were not excluded, the outcome may include prevalent or recurrent cases rather than purely incident cases.

This has implications for interpretation, particularly when describing “risk” or suggesting temporal direction. Clarification of how baseline CMD was handled would be helpful. If baseline cases were not excluded, this limitation should be explicitly acknowledged and reflected in the interpretation.

- Response: We thank the reviewer for this important clarification. We confirm that CMD at baseline was not assessed using diagnostic measures, and therefore participants with CMD at baseline were not excluded from the analyses. As a result, the outcome at follow-up may reflect a combination of incident, persistent, or recurrent cases rather than purely incident cases. In the revised manuscript, we have clarified this in both the Methods and Discussion sections. We have also revised the wording throughout to avoid implying that the results reflect incident risk only, and instead interpret the findings as associations with subsequent CMD status. This limitation has now been explicitly acknowledged and discussed.

4. Measurement of Physical Exercise
Physical exercise was assessed using self-reported measures of frequency, intensity, and duration, which are subject to recall and social desirability bias. In addition, the operationalisation of adherence to WHO recommendations appears relatively strict and may result in misclassification. A more explicit discussion of potential exposure misclassification and its likely direction of bias would strengthen the limitations section.

- Response: We thank the reviewer for this important point. We agree that the self-reported assessment of physical exercise may introduce measurement error, including both recall bias and social desirability bias. In addition, the operationalisation of adherence to WHO recommendations was relatively strict and may have led to some degree of misclassification.

In the revised manuscript, we have expanded the limitations section to explicitly address potential exposure misclassification and its likely direction. In general, such misclassification is likely to be non-differential with respect to subsequent CMD and would therefore tend to attenuate the observed associations. However, some degree of differential misclassification cannot be ruled out.

5. Causal Language and Interpretation
The manuscript occasionally uses language suggesting causality (e.g., “reducing risk”, “protective effect”), which may not be fully supported given the observational design and potential for residual confounding. More cautious wording that reflects associations rather than causal effects would improve the scientific accuracy of the interpretation.

- Response: We thank the reviewer for this important point. We agree that the observational design does not support causal inference and that some wording in the original manuscript may have implied causality. In the revised version, we have carefully reviewed the manuscript and replaced causal language (e.g., “reducing risk”, “protective effect”) with more appropriate terminology reflecting associations.
  Throughout the manuscript, we now consistently refer to “associations”, “lower relative risk”, or “lower likelihood” rather than implying causal effects. We have also clarified in the Discussion that residual confounding and reverse causation cannot be ruled out. These revisions improve the precision and scientific accuracy of the interpretation.

Minor Comments
There is some redundancy in the introduction, particularly in the description of physical exercise constructs (frequency, intensity, and duration), which appear more than once. Streamlining this section would improve clarity.

- Response: We thank the reviewer for this helpful observation. We have revised the Introduction to reduce redundancy, particularly in the description of physical exercise constructs (frequency, intensity, and duration). Repeated explanations have been streamlined and consolidated to improve clarity and flow, while retaining the necessary conceptual distinctions.

The description of inverse probability weighting and the “doubly robust” approach is technically sound but somewhat dense. Simplifying this section slightly may improve accessibility for a broader readership.

- Response: We thank the reviewer for this helpful comment. We have revised the Statistical Analyses section to improve clarity and accessibility. Specifically, we simplified the description of the inverse probability weighting procedure and provided a more intuitive explanation of the “doubly robust” approach, while retaining the essential methodological details. These changes aim to make the analytic strategy more transparent to a broader readership without altering the underlying methods.

The results are clearly presented, although some sections are somewhat verbose. Minor tightening could enhance readability without loss of content.

- Response: We thank the reviewer for this helpful observation. We have carefully reviewed the Results section and made minor revisions to improve conciseness and readability. Specifically, we reduced some redundancy and streamlined selected sentences, while ensuring that all key findings and details are retained.

The discussion is comprehensive but could be moderately shortened, as certain themes—particularly dose-response relationships and plateau effects—are repeated.

- Response: We thank the reviewer for this helpful suggestion. We have revised the Discussion section to improve conciseness by reducing repetition, particularly in relation to dose-response patterns and plateau effects. The text has been streamlined while retaining the key interpretations and context of the findings.

Potential residual confounding from factors such as mental health service use, psychotropic medication, and substance use is not discussed and should be acknowledged.

- Response: We thank the reviewer for this important suggestion. We agree that potential residual confounding from factors not measured in the present study should be acknowledged. In the revised manuscript, we have expanded the limitations section to note that unmeasured factors such as mental health service use, psychotropic medication, and substance use may influence both physical exercise and risk of CMD, and could therefore contribute to residual confounding. We clarify that, although we adjusted for several relevant sociodemographic variables and baseline psychological distress, these additional factors were not available and may have affected the observed associations.
