# Supplementary material for: Physical exercise and risk of common mental disorders among higher education students: A national prospective study
Source: PLoS One. 2026 Aug 19;21(8):e0353787. doi: 10.1371/journal.pone.0353787 (PMC13489442; doi:10.1371/journal.pone.0353787)
Supplement: Supplementary file 4 [file pone.0353787.s004.docx]

Reviewer #1

Reviewer #1: I was Reviewer 1 in the previous round. The authors addressed my comments very well, and I am satisfied with the revisions. I only have two very minor remaining comments:

**Response: We thank the reviewer for the positive assessment of the revised manuscript and for the careful reading of the revised version. We are pleased that the previous comments were satisfactorily addressed. We have revised the manuscript in response to the remaining minor suggestions as detailed below.**

On p. 10, the manuscript states: “Formal interaction analyses supported effect modification by sex for exercise frequency, particularly at the lowest frequency levels, whereas evidence for interaction by age group was limited and not consistent across exposure categories.”

a) I could not find a table reporting the formal interaction test results.

**Response: We agree that the original manuscript did not sufficiently report the formal interaction analyses. In response, we conducted formal interaction analyses within the same inverse probability weighted modified Poisson regression framework used in the main analyses.**

**Specifically, we tested sex × exercise frequency and age group × exercise frequency interaction terms in fully adjusted models including sociodemographic covariates and baseline psychological distress. The overall sex × exercise frequency interaction was statistically significant (Wald χ² = 14.91, df = 4, p = .005). Category-specific interaction terms indicated that sex differences were most evident at the two lowest exercise frequency levels, namely never/seldom exercise (p = .044) and exercise once per week (p = .034). In contrast, the overall age group × exercise frequency interaction was not statistically significant (Wald χ² = 6.71, df = 4, p = .152), although one category-specific term reached statistical significance. We have revised the Methods and Results sections to clarify that formal Wald interaction tests were conducted, and we now report the overall interaction tests directly in the manuscript. The stratified analyses are interpreted accordingly.**

b) The term “limited” is somewhat unclear. If the interaction effect was not statistically significant, I would recommend stating this more directly.

**Response: We agree and have revised the wording accordingly. In the revised analyses, the overall age group × exercise frequency interaction was not statistically significant (Wald χ² = 6.71, df = 4, p = .152). Although one category-specific interaction term reached statistical significance, the overall interaction test did not support meaningful effect modification by age group. We have therefore revised the manuscript to state this more directly and to avoid ambiguous wording such as “limited” evidence. The Results section now explicitly reports the overall interaction test and notes that the isolated significant category-specific finding should be interpreted cautiously in light of the non-significant overall interaction.**

c) Given the very large sample size, the nonsignificant interaction findings for age group do not seem to support the subsequent “age-stratified associations” section on p. 12. Some clarification of the rationale for presenting those stratified analyses would be helpful.
 **Response: We thank the reviewer for this important point. Age-stratified analyses were included because they were specified a priori based on previous literature suggesting that exercise and mental health associations may differ across developmental stages within higher education populations. However, we agree that these findings should be interpreted descriptively given the absence of a statistically significant overall age group × exercise frequency interaction. Although one category-specific interaction term reached statistical significance, the overall interaction test did not support meaningful age-related effect modification. We have therefore revised the manuscript to clarify that the age-stratified analyses are exploratory and descriptive in nature and should be interpreted cautiously.**

On p. 12, the statement “whereas the association in men were more nearly linear” is unclear. Does this mean that the quadratic effect was nonsignificant in men, or that both linear and quadratic effects were significant but the linear trend was predominant?

**Response: We have now revised the text to clarify that the quadratic term for weekly exercise hours was not statistically significant among men, indicating no statistical evidence of non-linearity. As shown in Supplementary Table S8, the quadratic term was statistically significant among women (p = .024), supporting a curvilinear association with diminishing returns at higher levels of exercise, whereas it was not statistically significant among men (p = .288). The manuscript now states explicitly that there was no statistical evidence of a non-linear association among men and that the observed association was adequately described by a linear trend.**

Reviewer #2

We thank the reviewer for the positive assessment of the revised manuscript and for the additional constructive suggestions. We are pleased that the reviewer considers the manuscript substantially improved. The points raised largely concern clarification and emphasis, and we have revised the manuscript accordingly.

First, although the prospective design strengthens temporal interpretation, the study remains observational and cannot establish causality. Some sections of the Discussion and Conclusion continue to imply that physical exercise prevents common mental disorders. I recommend adopting more cautious language throughout the manuscript and emphasizing that the findings demonstrate prospective associations rather than causal effects.

**Response: We agree and have undertaken an additional review of the manuscript to ensure that the language consistently reflects the observational nature of the study. We have revised selected passages throughout the Discussion and Conclusions to emphasise that the findings represent prospective associations with subsequent CMD rather than causal effects. In particular, we have avoided language implying that physical exercise prevents CMD and instead refer to lower likelihood, lower risk, or prospective associations with subsequent disorder status. We have also strengthened the discussion of residual confounding, reverse causation, and the inability of the present observational design to establish causality. The Conclusions section has been revised accordingly to emphasise physical exercise as a potential target for mental health promotion rather than as a proven preventive intervention.**

Second, the decision to combine major depressive episode and generalized anxiety disorder into a single CMD outcome is understandable and well justified. However, because depression and anxiety may have different relationships with physical exercise, additional discussion of this limitation would strengthen the interpretation of the findings. If data are available, supplementary analyses examining the outcomes separately would provide additional insight.

**Response: We agree and have further clarified this limitation in the Discussion. As described in the manuscript, we conducted supplementary analyses examining 30-day and 12-month generalized anxiety disorder and major depressive episode separately (Supplementary Tables S2-S5). These analyses showed broadly similar inverse patterns across outcomes, although associations were generally somewhat stronger and more consistent for generalized anxiety disorder than for major depressive episode. We now draw greater attention to these supplementary analyses in the Results and Discussion sections and explicitly acknowledge that the composite CMD outcome may obscure disorder-specific and temporal differences.**

Third, although inverse probability weighting was used to address differential follow-up, the relatively modest response rate at baseline and additional attrition during follow-up may still introduce selection bias. A more detailed discussion of the potential impact of non-response on the study findings would be beneficial.

**Response: We thank the reviewer for this suggestion. We have further expanded the limitations section to emphasise that, although inverse probability weighting was used to reduce bias arising from differential participation and oversampling, residual selection bias due to unmeasured factors cannot be excluded. We now clarify that selection may have occurred both at baseline and during the CIDI follow-up, and that individuals with more severe mental health problems may have been under-represented. We also state that the weighted estimates should be interpreted as reducing, but not eliminating, potential bias associated with non-response.**

Fourth, the physical exercise measures were self-reported and relatively crude, relying on frequency, intensity, duration, and total weekly hours. Further discussion of possible measurement error and misclassification, and how these may have influenced the observed dose-response relationships, would strengthen the limitations section.

**Response: We agree and have expanded the discussion of self-reported physical exercise measures. The revised manuscript now more explicitly acknowledges the potential for recall bias, social desirability bias, and exposure misclassification arising from relatively crude measures of frequency, intensity, duration, and total weekly hours. We also note that such misclassification would likely attenuate the observed associations and could therefore lead to underestimation of the dose-response gradients. However, differential misclassification cannot be entirely ruled out, particularly if reporting of exercise behaviour is related to underlying mental health.**

Fifth, the age-stratified findings are interesting and deserve further consideration. The stronger associations observed among younger students may have important implications for prevention. Expanding the discussion to explore possible explanations for these differences would add value to the manuscript.

**Response: We thank the reviewer for this thoughtful suggestion. We agree that the descriptively stronger associations among younger students may be informative and have added a brief discussion of possible explanations. Specifically, we note that younger students may experience greater behavioural variability, adjustment challenges during the transition into higher education, and heightened vulnerability to mental health difficulties, which could make regular exercise more strongly associated with subsequent CMD in this group. However, to avoid overinterpretation, we also emphasise that the overall age group × exercise frequency interaction was not statistically significant, and that the age-stratified findings should therefore be interpreted as descriptive and hypothesis-generating rather than evidence of clear age-related effect modification.**

Finally, the implications section would benefit from a more balanced discussion of practical challenges associated with promoting physical exercise among university students. Structural barriers such as academic workload, financial constraints, time limitations, and access to facilities should be acknowledged alongside recommendations for institutional approaches to promoting physical activity.

**Response: We agree and have expanded the implications section accordingly. The revised manuscript now provides a more balanced discussion of practical challenges associated with promoting physical exercise among students. Specifically, we acknowledge that sustained engagement in physical activity is shaped by structural barriers such as academic workload, time constraints, financial limitations, and access to suitable facilities. We further emphasise that institutional and environmental approaches, including accessible low-threshold programmes and opportunities for activity within academic schedules, may be necessary to support sustained engagement in physical activity within higher education settings.**

We thank the reviewer for the constructive comments and positive evaluation of the revised manuscript.
