# Supplementary material for: Physical exercise and risk of common mental disorders among higher education students: A national prospective study
Source: PLoS One. 2026 Aug 19;21(8):e0353787. doi: 10.1371/journal.pone.0353787 (PMC13489442; doi:10.1371/journal.pone.0353787)
Supplement: Peer review history

# Author comment: Author response to Decision Letter 0

**DOI:** 10.1371/journal.pone.0353787.r001

26 Jan 2026

Attachment Submitted filename: Response to Editorial Office.pdf

# Peer review: Decision Letter 0

**DOI:** 10.1371/journal.pone.0353787.r002

**Authors:** Juthatip Wiwattanapantuwong

18 Mar 2026

PONE-D-26-02304

Physical exercise and risk of common mental disorders among higher education students: A national prospective study

PLOS One

Dear Dr. Grasdalsmoen,

Thank you for submitting your manuscript to PLOS ONE. After careful consideration, we feel that it has merit but does not fully meet PLOS ONE’s publication criteria as it currently stands. Therefore, we invite you to submit a revised version of the manuscript that addresses the points raised during the review process.

Please submit your revised manuscript by May 02 2026 11:59PM. If you will need more time than this to complete your revisions, please reply to this message or contact the journal office at plosone@plos.org. When you're ready to submit your revision, log on to https://www.editorialmanager.com/pone/ and select the 'Submissions Needing Revision' folder to locate your manuscript file.

Please include the following items when submitting your revised manuscript:

- A letter that responds to each point raised by the academic editor and reviewer(s). You should upload this letter as a separate file labeled 'Response to Reviewers'.
- A marked-up copy of your manuscript that highlights changes made to the original version. You should upload this as a separate file labeled 'Revised Manuscript with Track Changes'.
- An unmarked version of your revised paper without tracked changes. You should upload this as a separate file labeled 'Manuscript'.

If you would like to make changes to your financial disclosure, please include your updated statement in your cover letter. Guidelines for resubmitting your figure files are available below the reviewer comments at the end of this letter.

If applicable, we recommend that you deposit your laboratory protocols in protocols.io to enhance the reproducibility of your results. Protocols.io assigns your protocol its own identifier (DOI) so that it can be cited independently in the future. For instructions see: https://journals.plos.org/plosone/s/submission-guidelines#loc-laboratory-protocols. Additionally, PLOS ONE offers an option for publishing peer-reviewed Lab Protocol articles, which describe protocols hosted on protocols.io. Read more information on sharing protocols at https://plos.org/protocols?utm_medium=editorial-email&utm_source=authorletters&utm_campaign=protocols.

We look forward to receiving your revised manuscript.

Kind regards,

Juthatip Wiwattanapantuwong

Guest Editor

PLOS One

**Journal Requirements:**

When submitting your revision, we need you to address these additional requirements.

1. Please ensure that your manuscript meets PLOS ONE's style requirements, including those for file naming. The PLOS ONE style templates can be found at

https://journals.plos.org/plosone/s/file?id=wjVg/PLOSOne_formatting_sample_main_body.pdf and

https://journals.plos.org/plosone/s/file?id=ba62/PLOSOne_formatting_sample_title_authors_affiliations.pdf

2. We note that the grant information you provided in the ‘Funding Information’ and ‘Financial Disclosure’ sections do not match.

When you resubmit, please ensure that you provide the correct grant numbers for the awards you received for your study in the ‘Funding Information’ section.

3. Please note that funding information should not appear in any section or other areas of your manuscript. We will only publish funding information present in the Funding Statement section of the online submission form. Please remove any funding-related text from the manuscript.

4. For studies involving third-party data, we encourage authors to share any data specific to their analyses that they can legally distribute. PLOS recognizes, however, that authors may be using third-party data they do not have the rights to share. When third-party data cannot be publicly shared, authors must provide all information necessary for interested researchers to apply to gain access to the data. (https://journals.plos.org/plosone/s/data-availability#loc-acceptable-data-access-restrictions)

For any third-party data that the authors cannot legally distribute, they should include the following information in their Data Availability Statement upon submission:

1) A description of the data set and the third-party source

2) If applicable, verification of permission to use the data set

3) Confirmation of whether the authors received any special privileges in accessing the data that other researchers would not have

4) All necessary contact information others would need to apply to gain access to the data

5. Please include a copy of Tables 1 and 2 which you refer to in your text on pages 8 and 9.

6. Please upload a copy of Supplementary Table S1 which you refer to in your text on page 10.

7. If the reviewer comments include a recommendation to cite specific previously published works, please review and evaluate these publications to determine whether they are relevant and should be cited. There is no requirement to cite these works unless the editor has indicated otherwise.

[Note: HTML markup is below. Please do not edit.]

Reviewers' comments:

Reviewer's Responses to Questions

**Comments to the Author**

1. Is the manuscript technically sound, and do the data support the conclusions?

The manuscript must describe a technically sound piece of scientific research with data that supports the conclusions. Experiments must have been conducted rigorously, with appropriate controls, replication, and sample sizes. The conclusions must be drawn appropriately based on the data presented.

Reviewer #1: Partly

Reviewer #2: Yes

**********

2. Has the statistical analysis been performed appropriately and rigorously?

Reviewer #1: No

Reviewer #2: Yes

**********

3. Have the authors made all data underlying the findings in their manuscript fully available?

The PLOS Data policy requires authors to make all data underlying the findings described in their manuscript fully available without restriction, with rare exception (please refer to the Data Availability Statement in the manuscript PDF file). The data should be provided as part of the manuscript or its supporting information, or deposited to a public repository. For example, in addition to summary statistics, the data points behind means, medians and variance measures should be available. If there are restrictions on publicly sharing data—e.g. participant privacy or use of data from a third party—those must be specified.

Reviewer #1: No

Reviewer #2: No

**********

4. Is the manuscript presented in an intelligible fashion and written in standard English?

PLOS ONE does not copyedit accepted manuscripts, so the language in submitted articles must be clear, correct, and unambiguous. Any typographical or grammatical errors should be corrected at revision, so please note any specific errors here.

Reviewer #1: Yes

Reviewer #2: Yes

**********

5. Review Comments to the Author

Please use the space provided to explain your answers to the questions above. You may also include additional comments for the author, including concerns about dual publication, research ethics, or publication ethics. (Please upload your review as an attachment if it exceeds 20,000 characters)

Reviewer #1: Review Comments

This study uses data from a national survey of university students in Norway to investigate the association between physical exercise and common mental disorders (CMD), defined as depression and anxiety disorders based on clinical criteria. As expected, the authors report associations between exercise indicators and CMD, and they also report differential effects by sex based on stratified analyses.

Overall, I recommend major revision before the manuscript can be considered for publication. I hope the authors can address the following concerns.

1. Motivation and contribution

The motivation of the study is not sufficiently developed. The authors emphasize that the outcome is based on clinically defined common mental disorders rather than symptom scales. However, it is not clear how this outcome substantially differs from commonly used mental health scales in terms of the implications for the research question. The association between physical exercise and mental health outcomes is already well established in the literature. Therefore, the manuscript should more clearly articulate how the present study advances the substantive literature. Even if PLOS ONE does not require highly novel findings, the authors should better justify the added value of this work.

2. Construction of the outcome variable

The definition of the outcome variable raises several concerns. If I understand correctly, CMD is defined as the presence of either major depressive episode (MDE) or generalized anxiety disorder (GAD) based on CIDI diagnostic criteria. In addition, diagnoses from two time frames (past 30 days or past 12 months) appear to be combined into a single binary outcome. Thus, multiple diagnostic definitions (MDE vs. GAD and 30-day vs. 12-month diagnoses) are effectively collapsed into a single CMD indicator using an OR rule.

This approach raises several conceptual and interpretational issues.

First, although diagnostic classifications are often treated as binary outcomes in epidemiological research, this approach inevitably reduces information about symptom severity and heterogeneity within disorders. While this may be acceptable depending on the research question, the potential loss of information should at least be acknowledged.

Second, combining two distinct disorders (depression and anxiety) and two temporal diagnostic definitions (30-day and 12-month) into a single outcome may obscure meaningful differences. These represent different clinical constructs and different time frames of disorder occurrence. As currently specified, it becomes difficult to determine whether the observed associations reflect depression, anxiety, recent episodes, or more distal 12-month diagnoses.

I would therefore recommend presenting additional analyses separating the diagnostic components (e.g., MDE vs. GAD, and possibly 30-day vs. 12-month diagnoses) to better clarify the pattern of associations. Such analyses would help readers understand whether the reported results are driven primarily by one disorder or one diagnostic time frame.

3. Treatment of exercise variables

The exercise variables are analyzed individually without controlling for other exercise-related variables. This approach makes interpretation difficult because different indicators of exercise (e.g., frequency, intensity, duration) are likely correlated.

For example, the analysis of meeting the MVPA criterion (>150 minutes/week) essentially compares individuals below and above the guideline threshold. However, this does not address whether meaningful differences exist around that threshold (e.g., between 120–150 minutes and 150–180 minutes per week).

A more informative analysis would examine:

• finer categories of weekly exercise hours and compare adjacent categories, or

• a continuous specification of exercise hours with a nonlinear or curvilinear term, allowing examination of the instantaneous rate of change around the guideline threshold.

Furthermore, the models examining frequency, intensity, and duration do not appear to control for each other. Because these variables are related, it is not surprising that they all show associations with the outcome. Joint models including multiple exercise indicators would provide more interpretable results.

4. Missing descriptive statistics

The manuscript does not provide a descriptive statistics table for the exercise variables. This omission is problematic because readers cannot understand the distribution of the key independent variables. At minimum, the manuscript should present summary statistics (means, standard deviations, and/or category frequencies) for all exercise measures.

5. Lack of exercise measures at follow-up

Exercise variables were measured only at Time 1, while CMD was measured at Time 2 approximately one year later. Without measuring exercise at Time 2, it is difficult to evaluate changes in behavior over time.

More importantly, the manuscript occasionally uses causal language (e.g., “reduce” or “protective factor”). Given the observational design and the absence of repeated measures of both exposure and outcome, such causal interpretations are not justified. In longitudinal research, stronger causal claims are typically supported by designs such as cross-lagged panel models with repeated measures of both constructs. At minimum, the authors should avoid causal language and interpret the findings more cautiously.

6. Justification of the one-year interval

The manuscript does not sufficiently justify the one-year interval between baseline and follow-up. The authors should explain why this time lag is theoretically or clinically meaningful for studying the relationship between exercise and the onset of CMD.

7. Literature review

The literature review is somewhat limited. A broader and more up-to-date review of the literature on physical exercise and mental health is needed. For example, a quick search reveals relevant studies such as Chiang et al. (2024) in JAMA Pediatrics that are not discussed. The manuscript would benefit from a more comprehensive review of recent work in this area.

8. Inconsistent statistical models

The main analyses use modified Poisson regression to estimate risk ratios, whereas the supplementary analyses for weekly exercise hours rely on logistic regression with conversion of predicted probabilities to relative risks. The authors should clarify the rationale for using different modeling approaches across analyses. It would also be helpful to report whether similar results are obtained if the continuous exposure analyses are estimated using the same modified Poisson framework as the primary models.

9. Testing sex and age differences

The manuscript reports separate analyses for men and women and for different age groups. However, stratified analyses alone do not formally test whether the associations differ across groups. A more standard approach would include interaction terms (e.g., sex × exercise variables or age group × exercise variables) to test whether the slopes differ significantly between groups.

10. Use of Python

The methods section states that analyses were performed in SPSS and Python. The role of Python in the analysis is not explained. The authors should clarify which analyses were conducted in Python and why this additional software was used.

11. Type of exercise

The manuscript focuses primarily on the amount of exercise (e.g., frequency, duration, and total weekly hours). It would also be informative to consider different types of exercise (for example, social vs. individual activities), as these may have different associations with mental health outcomes. I recognize that such information may not be available in the current dataset and therefore may not be addressable in the present study. Nevertheless, incorporating distinctions in exercise type in future research could provide a more nuanced understanding of the relationship between physical activity and mental health.

12. Editorial and submission issues

Several elements required for review appear to be missing, including page numbers, tables, and supplementary materials. This made it difficult to identify specific locations in the manuscript when preparing this review. Please ensure that all materials are included in future submissions.

13. Terminology consistency

The manuscript appears to use the terms prevalence and risk ratio somewhat interchangeably in the Results section. These concepts refer to different quantities and should be distinguished more clearly.

Specifically, prevalence refers to the proportion of participants with CMD at follow-up, whereas the regression models estimate risk ratios, which represent the relative probability of CMD between exposure groups. When describing descriptive statistics, it would be more appropriate to refer to prevalence. When interpreting the regression models, the text should consistently refer to risk ratios (or relative risks).

Clarifying this distinction would improve conceptual precision and avoid potential confusion for readers.

14. Interpretation of adjusted vs. unadjusted estimates

In the Results section (“Association between weekly exercise and CMD”), the authors state that “adjusted and unadjusted estimates were similar, suggesting limited confounding.” However, the curves in the figure appear different. The authors should clarify this interpretation and explain how similarity between adjusted and unadjusted estimates was assessed.

Reviewer #2: General Comments

This manuscript presents a large, national prospective cohort study examining the association between multiple dimensions of physical exercise and subsequent common mental disorders (CMD) among higher education students in Norway. The study addresses an important public health question and contributes meaningfully to the literature by utilising diagnostically defined outcomes (CIDI 5.0) rather than symptom-based measures.

Overall, the manuscript is well-written, methodologically rigorous, and highly relevant. The use of inverse probability weighting, stratified analyses, and multiple operationalisations of physical exercise are notable strengths. The findings are consistent with existing evidence and provide additional insight into dose-response relationships and the relative importance of exercise frequency.

However, several important methodological and interpretational issues require clarification, particularly regarding selection bias, causal interpretation, and the role of baseline psychological distress in the analytical framework.

Major Comments

1. Selection Bias and Representativeness

The analytic sample represents a relatively small proportion of the baseline cohort (10,460 of 53,362; ~17.6%). Although inverse probability weighting (IPW) was applied to address differential response, this approach relies on the assumption that all relevant predictors of non-response are measured and correctly specified.

The manuscript acknowledges minimal differences in observed variables; however, unmeasured factors (e.g., severity of mental illness, motivation, health behaviours) may still bias the results. The discussion would benefit from a more explicit acknowledgement of the limitations of IPW and the potential for residual bias arising from unmeasured factors. Where possible, further justification for the representativeness of the weighted sample would strengthen confidence in the findings.

2. Adjustment for Baseline Psychological Distress (HSCL-25)

The inclusion of baseline psychological distress (HSCL-25) in the fully adjusted model is an important but conceptually complex decision. HSCL-25 may act as a confounder but may also lie on the causal pathway between physical exercise and CMD. Adjusting for it may therefore attenuate the total effect and instead estimate a direct effect.

The manuscript would benefit from clearer articulation of the conceptual role of HSCL-25 within the analytical framework. Distinguishing between confounding and mediation, and interpreting Models 2 and 3 accordingly, would improve clarity. Explicit reference to an underlying causal framework (e.g., a directed acyclic graph) would further strengthen the justification.

3. Outcome Definition and Temporal Interpretation

CMD is defined as 30-day or 12-month MDE/GAD at follow-up. It is unclear whether participants with CMD at baseline were excluded from the analyses. If baseline cases were not excluded, the outcome may include prevalent or recurrent cases rather than purely incident cases.

This has implications for interpretation, particularly when describing “risk” or suggesting temporal direction. Clarification of how baseline CMD was handled would be helpful. If baseline cases were not excluded, this limitation should be explicitly acknowledged and reflected in the interpretation.

4. Measurement of Physical Exercise

Physical exercise was assessed using self-reported measures of frequency, intensity, and duration, which are subject to recall and social desirability bias. In addition, the operationalisation of adherence to WHO recommendations appears relatively strict and may result in misclassification.

A more explicit discussion of potential exposure misclassification and its likely direction of bias would strengthen the limitations section.

5. Causal Language and Interpretation

The manuscript occasionally uses language suggesting causality (e.g., “reducing risk”, “protective effect”), which may not be fully supported given the observational design and potential for residual confounding.

More cautious wording that reflects associations rather than causal effects would improve the scientific accuracy of the interpretation.

Minor Comments

There is some redundancy in the introduction, particularly in the description of physical exercise constructs (frequency, intensity, and duration), which appear more than once. Streamlining this section would improve clarity.

The description of inverse probability weighting and the “doubly robust” approach is technically sound but somewhat dense. Simplifying this section slightly may improve accessibility for a broader readership.

The results are clearly presented, although some sections are somewhat verbose. Minor tightening could enhance readability without loss of content.

The discussion is comprehensive but could be moderately shortened, as certain themes—particularly dose-response relationships and plateau effects—are repeated.

Potential residual confounding from factors such as mental health service use, psychotropic medication, and substance use is not discussed and should be acknowledged.

Strengths of the Study

The study has several important strengths, including a large national sample, prospective design, and the use of diagnostically defined outcomes via CIDI 5.0. The application of inverse probability weighting, examination of multiple dimensions of physical exercise, and inclusion of dose-response analyses enhance the robustness of the findings. The stratified analyses by sex and age add further depth, and the study has clear relevance for public health and student mental health policy.

Conclusion

This is a high-quality and important study that advances understanding of the relationship between physical exercise and clinically defined mental disorders in young adults. The manuscript is close to being suitable for publication, and addressing the issues related to selection bias, causal interpretation, and analytical clarity will further strengthen its contribution.

Recommendation

Minor Revision

**********

6. PLOS authors have the option to publish the peer review history of their article (what does this mean?). If published, this will include your full peer review and any attached files.

If you choose “no”, your identity will remain anonymous but your review may still be made public.

**Do you want your identity to be public for this peer review?**  For information about this choice, including consent withdrawal, please see our Privacy Policy.

Reviewer #1: No

Reviewer #2: No

**********

[NOTE: If reviewer comments were submitted as an attachment file, they will be attached to this email and accessible via the submission site. Please log into your account, locate the manuscript record, and check for the action link "View Attachments". If this link does not appear, there are no attachment files.]

To ensure your figures meet our technical requirements, please review our figure guidelines: https://journals.plos.org/plosone/s/figures

You may also use PLOS’s free figure tool, NAAS, to help you prepare publication quality figures: https://journals.plos.org/plosone/s/figures#loc-tools-for-figure-preparation.

NAAS will assess whether your figures meet our technical requirements by comparing each figure against our figure specifications.

# Author comment: Author response to Decision Letter 1

**DOI:** 10.1371/journal.pone.0353787.r003

5 May 2026

Please see the attached “Response to Reviewers” document for a detailed, point-by-point response.

Attachment Submitted filename: PA CIDI response letter.docx

# Peer review: Decision Letter 1

**DOI:** 10.1371/journal.pone.0353787.r004

**Authors:** Juthatip Wiwattanapantuwong

10 Jun 2026

PONE-D-26-02304R1

Physical exercise and risk of common mental disorders among higher education students: A national prospective study

PLOS One

Dear Dr. Grasdalsmoen,

Thank you for submitting your manuscript to PLOS ONE. After careful consideration, we feel that it has merit but does not fully meet PLOS ONE’s publication criteria as it currently stands. Therefore, we invite you to submit a revised version of the manuscript that addresses the points raised during the review process.

==============================

The manuscript shows substantial improvement in lines with comments from reviewer. Only some minor revisions required. Please see reviewers comments below.

==============================

Please submit your revised manuscript by Jul 25 2026 11:59PM. If you will need more time than this to complete your revisions, please reply to this message or contact the journal office at plosone@plos.org. When you're ready to submit your revision, log on to https://www.editorialmanager.com/pone/ and select the 'Submissions Needing Revision' folder to locate your manuscript file.

Please include the following items when submitting your revised manuscript:

- A letter that responds to each point raised by the academic editor and reviewer(s). You should upload this letter as a separate file labeled 'Response to Reviewers'.
- A marked-up copy of your manuscript that highlights changes made to the original version. You should upload this as a separate file labeled 'Revised Manuscript with Track Changes'.
- An unmarked version of your revised paper without tracked changes. You should upload this as a separate file labeled 'Manuscript'.

If you would like to make changes to your financial disclosure, please include your updated statement in your cover letter. Guidelines for resubmitting your figure files are available below the reviewer comments at the end of this letter.

If applicable, we recommend that you deposit your laboratory protocols in protocols.io to enhance the reproducibility of your results. Protocols.io assigns your protocol its own identifier (DOI) so that it can be cited independently in the future. For instructions see: https://journals.plos.org/plosone/s/submission-guidelines#loc-laboratory-protocols. Additionally, PLOS ONE offers an option for publishing peer-reviewed Lab Protocol articles, which describe protocols hosted on protocols.io. Read more information on sharing protocols at https://plos.org/protocols?utm_medium=editorial-email&utm_source=authorletters&utm_campaign=protocols.

As the corresponding author, your ORCID iD is verified in the submission system and will appear in the published article. PLOS supports the use of ORCID, and we encourage all coauthors to register for an ORCID iD and use it as well. Please encourage your coauthors to verify their ORCID iD within the submission system before final acceptance, as unverified ORCID iDs will not appear in the published article. *Only*  the individual author can complete the verification step; PLOS staff *cannot*  verify ORCID iDs on behalf of authors.

We look forward to receiving your revised manuscript.

Kind regards,

Juthatip Wiwattanapantuwong

Guest Editor

PLOS One

Journal Requirements:

1. If the reviewer comments include a recommendation to cite specific previously published works, please review and evaluate these publications to determine whether they are relevant and should be cited. There is no requirement to cite these works unless the editor has indicated otherwise.

2. Please review your reference list to ensure that it is complete and correct. If you have cited papers that have been retracted, please include the rationale for doing so in the manuscript text, or remove these references and replace them with relevant current references. Any changes to the reference list should be mentioned in the rebuttal letter that accompanies your revised manuscript. If you need to cite a retracted article, indicate the article’s retracted status in the References list and also include a citation and full reference for the retraction notice.

[Note: HTML markup is below. Please do not edit.]

Reviewers' comments:

Reviewer's Responses to Questions

**Comments to the Author**

1. If the authors have adequately addressed your comments raised in a previous round of review and you feel that this manuscript is now acceptable for publication, you may indicate that here to bypass the “Comments to the Author” section, enter your conflict of interest statement in the “Confidential to Editor” section, and submit your "Accept" recommendation.

Reviewer #1: All comments have been addressed

Reviewer #2: (No Response)

**********

2. Is the manuscript technically sound, and do the data support the conclusions?

The manuscript must describe a technically sound piece of scientific research with data that supports the conclusions. Experiments must have been conducted rigorously, with appropriate controls, replication, and sample sizes. The conclusions must be drawn appropriately based on the data presented.

Reviewer #1: Yes

Reviewer #2: Yes

**********

3. Has the statistical analysis been performed appropriately and rigorously?

Reviewer #1: Yes

Reviewer #2: Yes

**********

4. Have the authors made all data underlying the findings in their manuscript fully available?

The PLOS Data policy requires authors to make all data underlying the findings described in their manuscript fully available without restriction, with rare exception (please refer to the Data Availability Statement in the manuscript PDF file). The data should be provided as part of the manuscript or its supporting information, or deposited to a public repository. For example, in addition to summary statistics, the data points behind means, medians and variance measures should be available. If there are restrictions on publicly sharing data—e.g. participant privacy or use of data from a third party—those must be specified.

Reviewer #1: Yes

Reviewer #2: No

**********

5. Is the manuscript presented in an intelligible fashion and written in standard English?

PLOS ONE does not copyedit accepted manuscripts, so the language in submitted articles must be clear, correct, and unambiguous. Any typographical or grammatical errors should be corrected at revision, so please note any specific errors here.

Reviewer #1: Yes

Reviewer #2: (No Response)

**********

6. Review Comments to the Author

Please use the space provided to explain your answers to the questions above. You may also include additional comments for the author, including concerns about dual publication, research ethics, or publication ethics. (Please upload your review as an attachment if it exceeds 20,000 characters)

Reviewer #1: I was Reviewer 1 in the previous round. The authors addressed my comments very well, and I am satisfied with the revisions. I only have two very minor remaining comments:

On p. 10, the manuscript states: “Formal interaction analyses supported effect modification by sex for exercise frequency, particularly at the lowest frequency levels, whereas evidence for interaction by age group was limited and not consistent across exposure categories.”

a) I could not find a table reporting the formal interaction test results.

b) The term “limited” is somewhat unclear. If the interaction effect was not statistically significant, I would recommend stating this more directly.

c) Given the very large sample size, the nonsignificant interaction findings for age group do not seem to support the subsequent “age-stratified associations” section on p. 12. Some clarification of the rationale for presenting those stratified analyses would be helpful.

On p. 12, the statement “whereas the association in men were more nearly linear” is unclear. Does this mean that the quadratic effect was nonsignificant in men, or that both linear and quadratic effects were significant but the linear trend was predominant?

I would be happy to recommend acceptance of this paper. Please consider the minor suggestions above.

Reviewer #2: Dear Authors,

Thank you for your careful consideration of the previous review comments and for the revisions made to the manuscript. The revised version has improved in clarity, methodological transparency, and overall presentation. The study remains highly relevant and makes an important contribution to the literature on physical exercise and mental health among higher education students. The large national sample, prospective design, use of diagnostic outcomes based on the CIDI 5.0, and application of inverse probability weighting are notable strengths of the work.

While I appreciate the efforts made to address the earlier comments, I believe that several issues still warrant further attention before the manuscript is suitable for publication.

First, although the prospective design strengthens temporal interpretation, the study remains observational and cannot establish causality. Some sections of the Discussion and Conclusion continue to imply that physical exercise prevents common mental disorders. I recommend adopting more cautious language throughout the manuscript and emphasizing that the findings demonstrate prospective associations rather than causal effects.

Second, the decision to combine major depressive episode and generalized anxiety disorder into a single CMD outcome is understandable and well justified. However, because depression and anxiety may have different relationships with physical exercise, additional discussion of this limitation would strengthen the interpretation of the findings. If data are available, supplementary analyses examining the outcomes separately would provide additional insight.

Third, although inverse probability weighting was used to address differential follow-up, the relatively modest response rate at baseline and additional attrition during follow-up may still introduce selection bias. A more detailed discussion of the potential impact of non-response on the study findings would be beneficial.

Fourth, the physical exercise measures were self-reported and relatively crude, relying on frequency, intensity, duration, and total weekly hours. Further discussion of possible measurement error and misclassification, and how these may have influenced the observed dose-response relationships, would strengthen the limitations section.

Fifth, the age-stratified findings are interesting and deserve further consideration. The stronger associations observed among younger students may have important implications for prevention. Expanding the discussion to explore possible explanations for these differences would add value to the manuscript.

Finally, the implications section would benefit from a more balanced discussion of practical challenges associated with promoting physical exercise among university students. Structural barriers such as academic workload, financial constraints, time limitations, and access to facilities should be acknowledged alongside recommendations for institutional approaches to promoting physical activity.

Overall, I commend the authors for the revisions undertaken and believe the manuscript has improved substantially. Addressing the remaining points outlined above will further strengthen the scientific rigor, interpretation, and practical relevance of the study.

**********

7. PLOS authors have the option to publish the peer review history of their article (what does this mean?). If published, this will include your full peer review and any attached files.

If you choose “no”, your identity will remain anonymous but your review may still be made public.

**Do you want your identity to be public for this peer review?**  For information about this choice, including consent withdrawal, please see our Privacy Policy.

Reviewer #1: No

Reviewer #2: No

**********

[NOTE: If reviewer comments were submitted as an attachment file, they will be attached to this email and accessible via the submission site. Please log into your account, locate the manuscript record, and check for the action link "View Attachments". If this link does not appear, there are no attachment files.]

To ensure your figures meet our technical requirements, please review our figure guidelines: https://journals.plos.org/plosone/s/figures

You may also use PLOS’s free figure tool, NAAS, to help you prepare publication quality figures: https://journals.plos.org/plosone/s/figures#loc-tools-for-figure-preparation.

NAAS will assess whether your figures meet our technical requirements by comparing each figure against our figure specifications.

# Author comment: Author response to Decision Letter 2

**DOI:** 10.1371/journal.pone.0353787.r005

25 Jun 2026

Please see the uploaded "Response to Reviewers" document for our detailed responses to all reviewer comments.

Attachment Submitted filename: PONE-D-26-02304R1_Response_to_Reviewers.docx.docx

# Editor report: Decision Letter 2

**DOI:** 10.1371/journal.pone.0353787.r006

**Authors:** Juthatip Wiwattanapantuwong

29 Jun 2026

Physical exercise and risk of common mental disorders among higher education students: A national prospective study

PONE-D-26-02304R2

Dear Dr. Grasdalsmoen,

We’re pleased to inform you that your manuscript has been judged scientifically suitable for publication and will be formally accepted for publication once it meets all outstanding technical requirements.

Within one week, you’ll receive an e-mail detailing the required amendments. When these have been addressed, you’ll receive a formal acceptance letter and your manuscript will be scheduled for publication.

An invoice will be generated when your article is formally accepted. Please note, if your institution has a publishing partnership with PLOS and your article meets the relevant criteria, all or part of your publication costs will be covered. Please make sure your user information is up-to-date by logging into Editorial Manager at Editorial Manager® and clicking the ‘Update My Information' link at the top of the page. For questions related to billing, please contact billing support.

If your institution or institutions have a press office, please notify them about your upcoming paper to help maximize its impact. If they’ll be preparing press materials, please inform our press team as soon as possible -- no later than 48 hours after receiving the formal acceptance. Your manuscript will remain under strict press embargo until 2 pm Eastern Time on the date of publication. For more information, please contact onepress@plos.org.

Kind regards,

Juthatip Wiwattanapantuwong

Guest Editor

PLOS One

Additional Editor Comments (optional):

All comments from reviewers were addressed in this revision.

Reviewers' comments:

# Editor report: Acceptance letter

**DOI:** 10.1371/journal.pone.0353787.r007

**Authors:** Juthatip Wiwattanapantuwong

PONE-D-26-02304R2

PLOS One

Dear Dr. Grasdalsmoen,

I'm pleased to inform you that your manuscript has been deemed suitable for publication in PLOS One. Congratulations! Your manuscript is now being handed over to our production team.

At this stage, our production department will prepare your paper for publication. This includes ensuring the following:

* All references, tables, and figures are properly cited

* All relevant supporting information is included in the manuscript submission,

* There are no issues that prevent the paper from being properly typeset

You will receive further instructions from the production team, including instructions on how to review your proof when it is ready. Please keep in mind that we are working through a large volume of accepted articles, so please give us a few days to review your paper and let you know the next and final steps.

Lastly, if your institution or institutions have a press office, please let them know about your upcoming paper now to help maximize its impact. If they'll be preparing press materials, please inform our press team within the next 48 hours. Your manuscript will remain under strict press embargo until 2 pm Eastern Time on the date of publication. For more information, please contact onepress@plos.org.

You will receive an invoice from PLOS for your publication fee after your manuscript has reached the completed accept phase. If you receive an email requesting payment before acceptance or for any other service, this may be a phishing scheme. Learn how to identify phishing emails and protect your accounts at https://explore.plos.org/phishing.

If we can help with anything else, please email us at customercare@plos.org.

Thank you for submitting your work to PLOS ONE and supporting open access.

Kind regards,

PLOS ONE Editorial Office Staff

on behalf of

Dr. Juthatip Wiwattanapantuwong

Guest Editor

PLOS One
